# High-Glucose Inhibits Human Fibroblast Cell Migration in Wound Healing via Repression of bFGF-Regulating JNK Phosphorylation

**DOI:** 10.1371/journal.pone.0108182

**Published:** 2014-09-22

**Authors:** Yuan Hu Xuan, Bin Bin Huang, Hai Shan Tian, Li Sha Chi, Yuan Meng Duan, Xi Wang, Zhong Xin Zhu, Wan Hui Cai, Yu Ting Zhu, Tie Min Wei, Hong Bo Ye, Wei Tao Cong, Li Tai Jin

**Affiliations:** 1 School of Pharmaceutical Sciences, Key Laboratory of Biotechnology Pharmaceutical Engineering, Wenzhou Medical University, Wenzhou, China; 2 The Third Affiliated Hospital of the Wenzhou Medical University, Wenzhou, China; 3 The Affiliated Wenling Hospital of Wenzhou Medical University, Wenling, China; 4 The Fifth Affiliated Hospital of Wenzhou Medical University, Lishui, China; 5 Department of Biology, Stanford University, Stanford, California, United States of America; National Centre for Scientific Research, ‘Demokritos’, Greece

## Abstract

One of the major symptoms of diabetes mellitus (DM) is delayed wound healing, which affects large populations of patients worldwide. However, the underlying mechanism behind this illness remains elusive. Skin wound healing requires a series of coordinated processes, including fibroblast cell proliferation and migration. Here, we simulate DM by application of high glucose (HG) in human foreskin primary fibroblast cells to analyze the molecular mechanism of DM effects on wound healing. The results indicate that HG, at a concentration of 30 mM, delay cell migration, but not cell proliferation. bFGF is known to promote cell migration that partially rescues HG effects on cell migration. Molecular and cell biology studies demonstrated that HG enhanced ROS production and repressed JNK phosphorylation, but did not affect Rac1 activity. JNK and Rac1 activation were known to be important for bFGF regulated cell migration. To further confirm DM effects on skin repair, a type 1 diabetic rat model was established, and we observed the efficacy of bFGF on both normal and diabetic rat skin repair. Furthermore, proteomic studies identified an increase of Annexin A2 protein nitration in HG-stressed fibroblasts and the nitration was protected by activation of bFGF signaling. Treatment with FGFR1 and JNK inhibitors delayed cell migration and increased Annexin A2 nitration levels, indicating that Annexin A2 nitration is modulated by bFGF signaling via activation of JNK. Together with these results, our data suggests that the HG-mediated delay of cell migration is linked to the inhibition of bFGF signaling, specifically through JNK suppression.

## Introduction

Diabetes mellitus (DM) is a group of metabolic disorders that is one of the most significant diseases in the developed world, affecting more than 170 million people. A major symptom of DM is unfit hyperglycemia, which leads to severe complications. One of the complications in clinical medicine is impaired wound healing in approximately 15% of DM patients [1]. High blood sugar that is linked to the inhibition of wound healing by altering angiogenesis [2] and fibroblast cell migration in diabetic mice was 75% less common than in normoglycemic mice [3], but the underlying mechanism is still unknown.

Wound healing requires the coordination of several cell types including keratinocytes, fibroblasts, endothelial cells, macrophages and platelets. The process involves cell proliferation and migration, collagen deposition and remodeling, wound contraction and angiogenesis. Fibroblasts are the most important cells involved in producing and remodeling the extracellular matrix, and fibroblast cell proliferation and migration play key roles in the formation of granulation tissue and further wound repair [4], [5]. Cell migration consisting of a multi-step cyclic process is necessary for wound repair. The basic migration pattern requires extension of a protrusion, stable attachment to near the leading edge of the protrusion, forward movement of the cell body and release of adhesions and retraction at the cell rear [6]–[8]. Rho family GTPases and actin proteins are key regulators in cell migration steps. Rac1 activates the actin-mediated Wave complex to induce the formation of lamellipodial protrusions at the leading edge of migrating cells [9], and Cdc42 regulates the polarity of migrating cells [10]. RhoA is activated in the rear and front of migrating cells to promote the contraction of actin stress fibers to generate contractile forces that cooperate with Rac1 and Cdc42 to induce membrane ruffles [11]–[14].

Wound healing processes are regulated by numerous growth factors. bFGF is a well-known member of the FGF family protein that modulates the growth, differentiation, migration and survival of a wide variety of cell types [15]. bFGF binds to the extracellular region of FGF receptor (FGFR) to activate downstream components, like Ras/MAPK which play roles in cell proliferation [16]. bFGF also regulates the PI3K-Rac1-JNK pathway to promote fibroblast cell migration [5], and increases expression of fibronectin, but not of collagen, in human fibroblasts [17]. A recent study of HG effects on a few different cell types, including fibroblast cells, has shown that HG-induced oxidative stress abnormally activates a key bFGF signaling protein Rac1 to delay cell migration [18]. Additionally, bFGF was reported to promote skin regeneration in diabetic rats [19]. Fibroblasts are some of the major targets of bFGF in wound healing; therefore, many studies have been published regarding the efficacy of bFGF. However, there are fewer reports that describe the molecular basis for the relationship between diabetes mellitus and bFGF.

In addition, the tissue responses to diabetic conditions are different, but many of them are associated with oxidative stress [20]. Mechanistic studies on DM effects have revealed that major causes and complications of DM are oxidative stress and nitrations of its mediated protein [21]–[23]. However, the events of protein nitration in fibroblasts under diabetic conditions have not been reported. In this study, human foreskin fibroblasts were utilized to analyze DM effects on wound healing and the efficacy of bFGF on the protection of DM-mediated delay of cell migration. bFGF activated JNK phosphorylation in both normal and HG-fed cells and rescued delayed cell migration caused by HG. Interestingly, we identified an increase of Annexin A2 nitration in HG-stressed fibroblast cells. A similar phenomenon was also observed in the cells with supplementation of FGFR1 and JNK inhibitors. Therefore, the data implies that Annexin A2 nitration that is modulated by bFGF signaling via JNK activity. Together with these results, our data suggests that the HG-mediated delay of cell migration is linked to the inhibition of bFGF signaling, specifically through JNK suppression.

## Materials and Methods

### 1. Ethics Statement

All animals were from the Laboratory Animals Center of Wenzhou Medical University, and treated strictly in accordance with international ethical guidelines and the National Institutes of Health's Guide for the Care and Use of Laboratory Animals. The experiments were carried out with the approval of the Animal Experimentation Ethics Committee of Wenzhou Medical University.

Human foreskin samples were collected from volunteers in the Second Affiliated Hospital, Wenzhou Medical University. All volunteers were informed of the purpose and procedure of this study and agreed to offer their tissue specimens. Written consent was obtained from all participants involved in this study. All protocols were approved by the Ethics Committee of the Second Affiliated Hospital of Wenzhou Medical University, Wenzhou, China.

### 2. Human foreskin fibroblast cell culture

All fat was removed from the human foreskin samples and the tissue was cut into 3 mm strips and incubated in Dispase (0.05%) and DMEM (Hyclone) at 4°C overnight. The epidermis was subsequently removed from the dermis. The dermis was minced finely and placed appropriately in 25 cm^2^ flasks (in advance-coated with FBS, Hyclone), and placed horizontally for one hour and then vertically for three hours in an atmosphere of 5% CO_2_ at 37°C. The tissues were cultured in DMEM, which contained 5.5 mM glucose, 10% FBS and 1% penicillin-streptomycin (Gibco), with subsequent changes of the medium every 3 days. The cultured cells were digested and passaged with 0.25% trypsin (Gibco) after the cell confluence reached approximately 80%. Cells passaged 3–6 times were selected for the following experiments. The human foreskin fibroblasts (HSFs) were treated with 5.5 mM glucose, 30 mM glucose or 24.5 mM mannitol together with 5.5 mM glucose for three days [24], [25].

### 3. Cell proliferation assay

Cell proliferation was assayed using a CCK-8 Kit (Dojindo Bio., Japan). In summary, 100 µL of cell resuspension solution (2×10^3^ cells/well) were transferred into 96-well plates after digestion with trypsin, and five parallel wells were used for each treatment. After attachment, the cells were subjected to the different treatments and then cultured for 72 hours in a 5% CO_2_ incubator at 37°C. Subsequently, 10 µL of CCK-8 was added into each well, and the cells were cultured for another 3 hours. Cell density was determined by measuring the absorbance at 450 nm using a Varioskan Flash (Thermo Scientific, USA). For analyzing HG effects on cell proliferation, 2000 cells were plated in 96 well plates and then the media were transferred, containing either normal (10%) or low (0.35%) fetal bovine serum (FBS) with different concentrations of glucose. After a 3-day incubation period, CCK-8 was added, and cell OD was measured. Different concentrations of bFGF were added after 3 days of HG (0.5% FBS) incubation to analyze the efficacy. Cell densities were measured 48, 72 and 96 hours after bFGF addition [24].

### 4. Wound healing assay

Cell migration was determined using the wound healing scratch assay as previously described [5], [26]. Cells were seeded on a 3.5 cm dish and grown overnight. Confluent cells were cultured in DMEM containing 0.5% FBS treated with 5 µg/mL mitomycin-C. After 3 days incubation in LG or HG media, cells were transferred to the medium containing low FBS (0.5%) and 5 µg/mL mitomycin-C. Effects of HG, bFGF (100 ng/mL) and FGFR1 inhibitor PD173074 (50 nM) on wound healing were measured after 12, 24, 36 and 48 hours. Images of the wounded cell monolayers were taken using a microscope (model IX70; Olympus, Tokyo, Japan) at 0, 12, 24, 36 and 48 hours after wounding and recorded for 48 hours using a microscope (model IX-70; Olympus) equipped with a CCD Camera (CoolSNAP HQ; Nippon Roper, Chiba, Japan) and controlled by MetaMorph software (Universal Imaging Co., Ltd., UK). All experiments were performed in the presence of 5 µg/mL of mitomycin-C to inhibit cell proliferation.

### 5. Rac1 pull-down assay

Measurement of Rac1 activity was performed according to the modified method as described previously [5]. In brief, the cells were scraped into an ice-cold lysis buffer containing 50 mM Tris-HCl (pH 7.4), 2 mM MgCl_2_, 1% NP40, 10% glycerol, 100 mM NaCl and Protease Inhibitor Cocktail (Roche Diagnostics, Basel, Switzerland), and centrifuged for 15 min at 16,000 g. The cleared lysates were incubated with 20 µg PAK-1 PBD agarose (Thermo, USA) or 20 µg GST-tagged Rhotekin Rho binding domain bound to glutathione agarose for 60 min at 4°C. The beads were washed three times with a lysis buffer and heated for 5 min at 100°C in the reducing SDS-PAGE sample buffer, and then analyzed for bound Rac1 molecules by Western blot analysis using an anti-Rac1 mouse monoclonal antibody (1∶1000).

### 6. Measurement of reactive oxygen species (ROS)

ROS levels were measured with the 2′, 7′-dichlorofluorescein diacetate (DCFH-DA), as previously described [27]. HSF samples were treated with HG for 72 hours before treating with bFGF (100 ng/ml) or PD173074 for 1 hour. Then the cells were washed three times with PBS. DCFH-DA, diluted to a final concentration of 10 µM, was added to the HSFs, which subsequently were incubated for 30 min at 37°C in the dark. After the cells were washed three times with serum-free medium, the fluorescence intensity was detected with a multi-detection microplate reader with excitation at 488 nm and emission at 530 nm within 15 min. Control cells were treated in the same way except adding bFGF and PD173074 to the culture medium. The fluorescence values were measured by using Image-Pro plus software. In addition, cells were treated in the same way except adding bFGF and SP600125 to the culture medium.

Dihydroethidium (DHE) staining was used to evaluate the contents of superoxide in situ. Rat skin tissues were embedded in an optical cutting temperature (OCT) compound to freeze at -20°C. The tissue was cut into 10 µm sections to which DHE (2×10^−6^ mol/L) was immediately applied at 37°C for 30 min in the dark. Images were obtained by fluorescence microscopy (Nikon, Japan, Tokyo) using an excitation wavelength of 488 nm and an emission wavelength of 610 nm [28]. Fluorescence was digitally quantified by using a software package (Image Pro Plus version 6.0, USA).

### 7. Creation of skin wounds on diabetic rats and treatment with bFGF

A type 1 diabetic rat model was established as described previously [19]. Briefly, male SD rats, weighting 180–220 g, were given a single intraperitoneal injection of 60 mg/kg STZ dissolved in the sodium citrate buffer (pH 4.5). Rats serving as controls were given the same volume of sodium citrate. STZ-treated mice with glucose levels>16.7 mmol/L were considered as being diabetes [29]. After being anesthetized with pentobarbital (45 mg/mL), the dorsal area of diabetic rats was totally depilated by Na_2_S (8.0%, w/v) and two full-thickness circular wounds (about 250 mm^2^ each) were created on the lower back of each rat using a pair of sharp scissors and a scalpel. bFGF (100 ng/mL) was applied to the skin around the diabetic skin wounds.

### 8. Microscopic evaluation of wound healing

The wound area was examined after treatment for 0, 4, 8, 12 and 16 days. The skin wounds of three rats were photographed, and the unhealed wound area was measured based on images using the TINA 2.0 software [19].

### 9. Western blot analysis

The cells cultured in the presence of 5 µg/ml of mitomycin-C were lysed in a RIPA buffer containing 1 mM Na_3_VO_4_, 1 mM NaF and Protease Inhibitor Cocktail (Roche Diagnostics, Basel, Switzerland), incubated for 20 min at 4°C and centrifuged at 15,000 g for 15 min at 4°C. The proteins were separated on SDS-PAGE and electrotransferred onto Immobilon-P Transfer Membranes (MILLIPORE JAPAN, Tokyo, Japan). The membranes were incubated in TBS containing 5% skim milk and 0.05% Tween-20 for 60 min and blotted with primary antibodies at 4°C overnight. An anti-p53 antibody (1∶2000, Abcam, Cambridge, USA), anti-c-Casepase-3 antibody (1∶2000, Abcam, Cambridge, USA), anti-PAI-1 antibody (1∶2000, Abcam, Cambridge, USA), anti-TNF-α antibody (1∶2000, Abcam, Cambridge, USA), anti-phospho-AKT antibody (1∶1000, Cell Signaling Technology, Massachusetts, USA), anti-AKT antibody (1∶1000, Cell Signaling Technology), anti-phospho-JNK antibody (1∶1000, Cell Signaling Technology), anti-JNK antibody (1∶1000, Cell Signaling Technology), anti-3-nitrotyrosine antibody (1∶2000, Abcam, Cambridge, USA), anti-Annexin A2 antibody (1∶1000, Abcam, Cambridge, USA), and anti-GAPDH antibody (1∶2000, Abcam, Cambridge, USA) were used as primary antibodies. The membranes were incubated for 1 hour with an anti-mouse or anti-rabbit HRP-linked secondary antibody (1∶2000, Cell Signaling Technology). Reaction products were visualized by detection of chemiluminescence using an ECL Western Blotting Detection System (GE Healthcare, Piscataway, NJ, USA). Quantification of relative band densities was performed by scanning densitometry using Image J software (National Institute of Health, Bethesda, MD, USA).

### 10. Electroelution

The whole gel eluter (Bio-Rad) was used according to the manufacturer's instructions. Briefly, 40 bands of Annexin A2 (∼36–40 kDa) were electro-eluted from the gel. The gel eluter apparatus was filled with the elution buffer (25 mM Tris, 0.25 M Glycine, 0.1% SDS). Proteins were transferred to the unit at 250 mA for 60 min. Protein fractions (1.5 mL for each) were harvested and concentrated by vacuum centrifugation to a volume of 200 µL. Then the protein samples were mixed with 600 µL of ice-cold acetone, stored for 2 hours at −20°C, and centrifuged at 14,000 g for 10 min. The supernatant was carefully removed and discarded. Protein pellets were dried by vacuum centrifugation.

### 11. In-gel digestion

Samples were first separated on 10.6% SDS polyacrylamide gel and stained with ethyl-violet and zincon [30]. Gel spots of interest were excised from the gels, cut into small pieces, and extensively soaked in a destaining solution (10% acetic acid, 30% ethanol) for at least 3 hours. Between steps, gel slices were dehydrated using acetonitrile. After in-gel reduction (DTT 10 mM in final, 45 min, 56°C) and alkylation (IAA 55 mM in final, 30 min in the dark), gel slices were recovered by trypsin (Promega, Madison, WI, USA) at 12.5 ng/µL in 25 mM ammonium bicarbonate, 5 mM CaCl_2_ on ice for 45 min. After rehydration of gel slices with this trypsin solution, the excess of solution was removed and gel slices were incubated overnight at 37°C. Peptides were extracted from gel slices and incubated for 15 min in 25 mM ammonium bicarbonate and 15 min in 5% formic acid. Supernatants from all fractions of the same sample were pooled and dried using a SpeedVac. Samples were desalted on a ZipTip C18 column (Millipore) according to the manufacturer's instructions and dried using a SpeedVac. Then formic acid was added into samples to yield a final content of 0.1% (v/v), which were finally analyzed by mass spectrometry.

### 12. Nanoelectrospray ionization-tandem mass spectrometry

In-gel tryptic digests (4 µL) were submitted to on-line Nanoflow liquid chromatography using the EASY-nLC system (Proxeon Biosystems, Odense, Denmark, now part of Thermo Fisher Scientific) with 10 cm capillary columns of an internal diameter of 75 µm filled with 3 µm Reprosil-Pur C_18_-A2 resin (Dr. Maisch GmbH, Ammerbuch-Entringen, Germany). The gradient consisted of 10–30% acetonitrile in 0.1% formic acid at a flow rate of 200 nL/min for 45 min, 30–100% acetonitrile in 0.1% formic acid at a flow rate of 200 nL/min for 1 minute, and 100% acetonitrile in 0.1% formic acid at a flow rate of 200 nL/min for 10 min. The eluter was electrosprayed through a Proxeon nanoelectrospray ion source by ESI-MS/MS analysis on a Thermo Fisher LTQ Velos Pro (Thermo Fisher Scientific, Bremen, Germany), using full ion scan mode over the m/z range 200–1800. Collision-induced dissociation (CID) was performed in the linear ion trap using a 4.0-Th isolation width and 35% normalized collision energy with helium as the collision gas. Five dependent MS/MS scans were performed on each ion using dynamic exclusion. The precursor ion that had been selected for CID was dynamically excluded from further MS/MS analysis for 30 sec.

### 13. Data process and database search

The MS/MS spectra were processed using Proteome Discoverer (Version 1.4, Thermo Fisher Scientific-Waltham, USA) and the database search was performed using the Mascot search engine (Matrix Science Mascot 2.4). We searched the Swiss-Prot protein sequence database (release 54.5), with the taxonomy selection set at *Homo sapiens* as an example. The search parameters were the following: mass error tolerance for the precursor ions 1.5 Da; mass error tolerance for the fragment ions, 0.8 Da; fixed modifications, carbamidomethylation (C); variable modifications, oxidation (M), nitration (Y, W); number of missed cleavages, 2; significance threshold, *p<0.05*; type of instrument, ESI-TRAP. Protein identifications were validated only if they met the following 3 requirements: (a) their score was significant (*p<0.05*) with cut-off criteria; (b) they were identified with one peptide with a score>35 or two peptides with score>30; (c) they were identified in at least two out of the three runs. Proteins identified by a set or subset of peptides used for identification of another protein were not taken into account.

### 14. Statistical analysis

Statistical calculations were performed by prism 5 (GraphPad, San Diego, CA). All data were expressed as mean ±SE. Comparisons between the two groups were performed by a *t* test.

## Results

### 1. bFGF reverses HG-induced apoptosis and inflammation in fibroblasts

**Figure 1:**
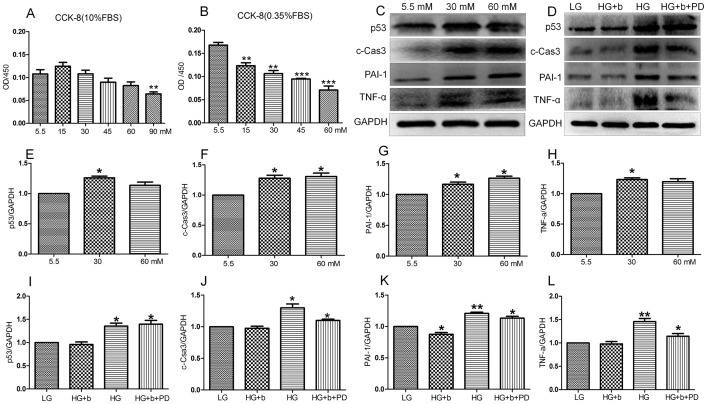
The protection of bFGF on HG-stimulated inflammation and apoptosis. Cell proliferation under HG conditions with 10% (A) or 0.35% (B) FBS (fetal bovine serum) was measured by CCK-8 assay after a 72-hour incubation (**P<0.01, ***P<0.001, *t* test). (C) Immunoblotting analysis for detection of p53, c-Caspase-3, PAI-1 and TNF-α in HSFs treated with the indicated concentrations of glucose for 72 hours. (D) HSFs were pretreated with 30 mM glucose (HG) for 72 hours and then incubated with 100 ng/mL bFGF (b) or bFGF together with FGFR1 inhibitor PD173074 (PD, 50 nM) for another 1 hour. The expression of levels of TNF-α, PAI-1, c-Caspase-3and p53 were analyzed by immunoblotting. LG means 5.5 mM glucose in culture medium and GAPDH was used as an internal control. Densitometry for p53 (E) or c-Caspase-3 (F) or PAI-1 (G) or TNF-α (H) shown in (C) was normalized to the amount of GAPDH. Densitometry for p53 (I) or c-Caspase-3 (J) or PAI-1 (K) or TNF-α (L) shown in (D) was normalized to the amount of GAPDH. The results are presented as fold change as compared with fibroblasts grown in the 5.5 mM(LG)glucose containing medium. Data represent mean values ±SE of three independent experiments (*P<0.05, *t* test).

As a simulation of diabetes, HG was used to study its effects on wound healing [18]. As a step before testing the effects of HG on wound healing, cell proliferation was monitored using different concentrations of glucose and FBS (fetal bovine serum) in the culture medium. In 10% FBS, HG (up to 60 mM) did not obviously change cell proliferation, while 90 mM glucose inhibited cell proliferation compared to a low glucose (LG, 5.5 mM) condition (Figure 1A). On the other hand, HG drastically affected cell proliferation, even with a concentration of 15 mM in a 0.35% FBS containing medium (Figure 1B). As shown in Figure 1A, HG with 10% FBS in the growth medium did not affect cell proliferation activity, so further biochemical studies examined whether HG damaged cells. Two apoptosis and inflammation (TNF-α and PAI-1) marker proteins were isolated to examine the effects of HG on fibroblasts. The results showed that HG (30 and 60 mM)-induced protein levels of p53, c-Caspase-3, TNF-α and PAI-1 after a 72-hour stimulation (Figure 1C, E, F, G, H). bFGF as a member of the FGF family has been shown to protect the heart from injury associated with a heart attack, and reduces tissue death [31]. Therefore, the role of bFGF in the protection of HG (30 mM)-induced damage in fibroblast cells was further analyzed. bFGF application leads to suppression of HG-mediated overexpression of p53, c-Caspase-3, TNF-α and PAI-1, but supplementation of PD173074 (PD, 50 nM) FGFR1 inhibitor together with bFGF diminished efficacy of bFGF to protect HG damage to the fibroblasts (Figure 1D, I, J, K, L). These results suggested that HG triggered cellular damage, but it did not change cell proliferation activity with 10% FBS in the culture medium. bFGF eliminated HG induced damage via activation of the FGFR1-mediated signaling pathway in human fibroblasts.

### 2. HG impairs fibroblast cell migration via inhibition of bFGF-regulated JNK phosphorylation

Previous studies reported that bFGF promotes fibroblast proliferation, and proliferation is one of the key processes in wound healing [32]. To test the bFGF effects on cell proliferation of fibroblasts grown in HG medium, different concentrations of bFGF (50–400 ng/mL) were treated to cells that were grown in a 30 mM glucose-containing medium. Cell numbers were counted using a CCK-8 kit 48, 72 and 96 hours after adding bFGF (Figure S1). The results indicated that bFGF promotes cell proliferation of HG stressed fibroblast cells. Therefore, it becomes necessary to exclude the effects on proliferation when evaluating bFGF-induced cell migration in wound healing. Mitomycin-C has been introduced to block cell proliferation without damaging cells at a concentration of 5 µg/mL [5]. We further examined the effects of different concentrations of mitomycin-C on human foreskin fibroblasts and observed that 5 µg/mL of mitomycin-C was the optimal concentration (data not shown).

**Figure 2:**
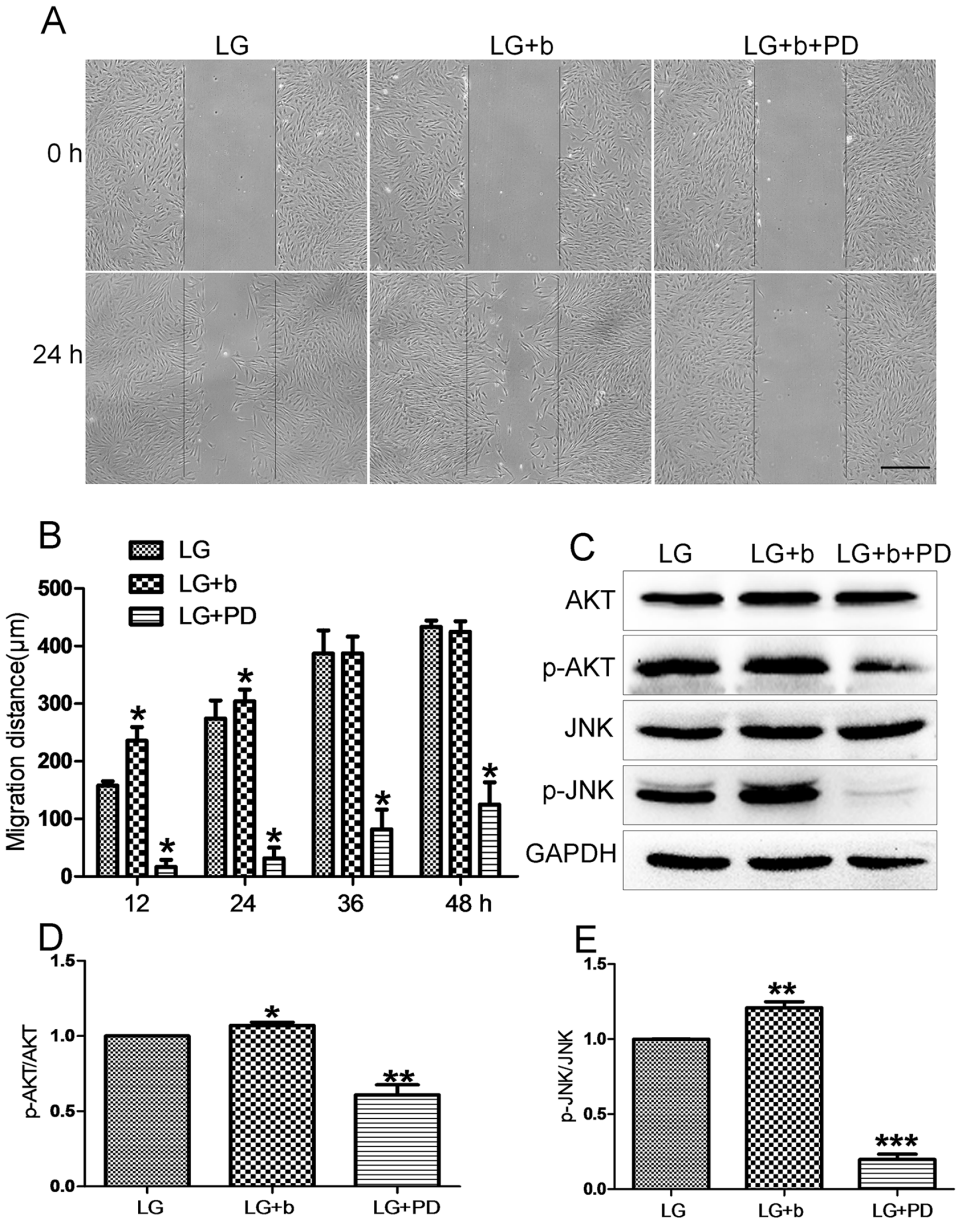
Effects of inhibition of FGFR1 on fibroblast migration and JNK phosphorylation. (A) Wound healing assay was performed to analyzed effects of 50 nM of FGFR1 inhibitor PD173074 (PD) on human fibroblast cell migration and LG means 5.5 mM glucose in the culture medium. (B) Cell migration distance was measured according to the data shown in (A). Data represent mean values ±SE of 10 replicates, as compared to the HG group (*P<0.001, *t* test). (C) Phosphorylation levels of AKT and JNK were analyzed after 30 min of bFGF (b) and PD173074 (PD) stimulation. All experiments were performed after 5 µg/mL mitomycin-C (cell proliferation inhibitor) application for one day. Densitometry for p-AKT (D) and p-JNK (E) was normalized to the amount of total AKT and JNK. The results are presented as fold change as compared to fibroblasts grown in the 5.5 mM glucose (LG) containing medium. Data represent mean values ±SE of three independent experiments (*P<0.05, *t* test).

**Figure 3:**
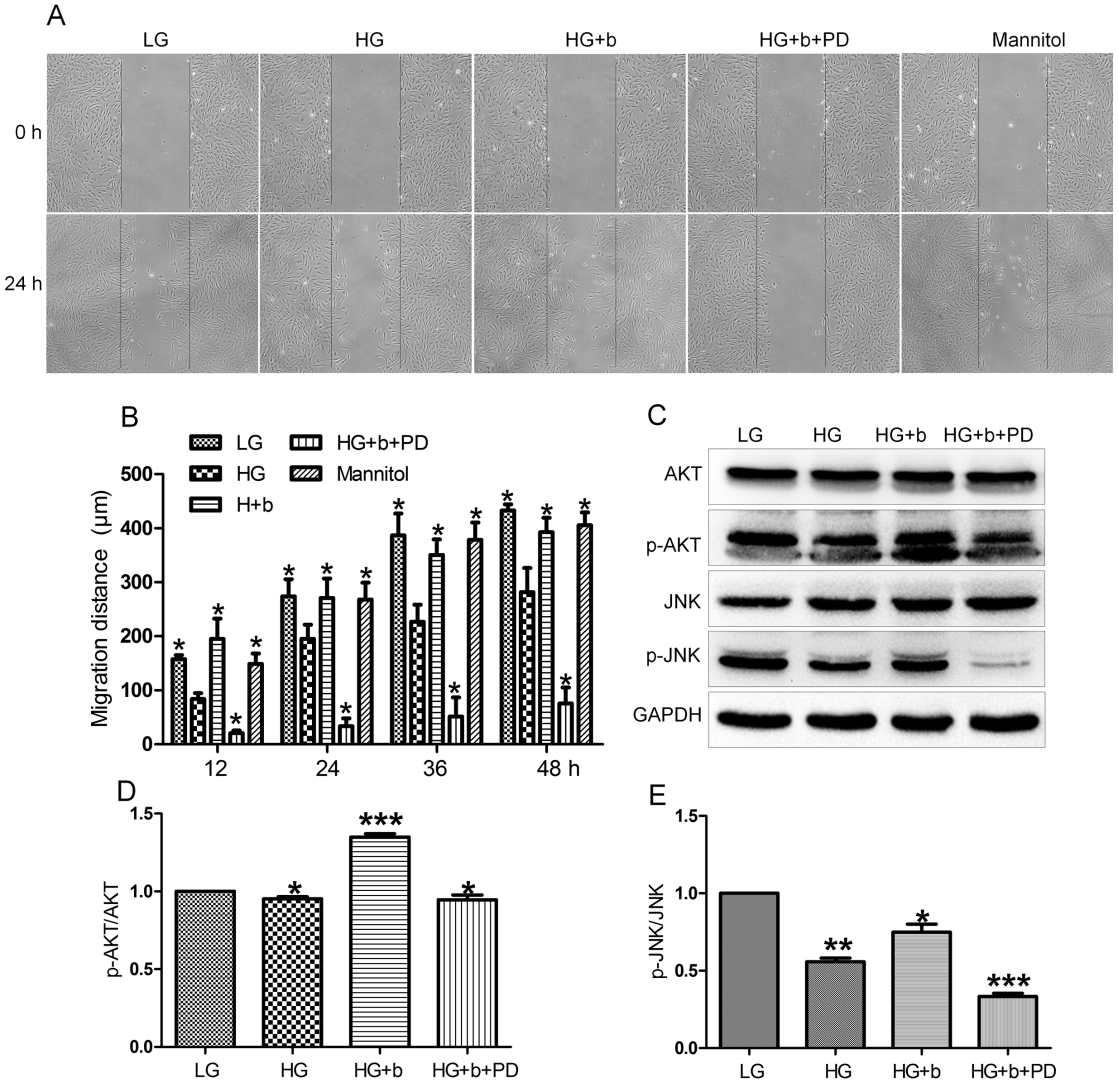
Effects of high glucose, bFGF and FGFR1 inhibitor on fibroblast cell migration and JNK phosphorylation. (A) Wound healing assay was performed to analyze effects of HG, 100 ng/mL of bFGF (b) and 50 nM of FGFR1 inhibitor PD173074 (PD) in human fibroblasts and mannitol was used as an osmotic control (5.5 mM glucose plus 24.5 mM mannitol) and LG means 5.5 mM glucose in the culture medium. (B) Cell migration distance was measured according to the data shown in (A). Data represent mean values ±SE of 10 replicates, as compared to the HG group (*P<0.001, *t* test). (C) Phosphorylation levels of AKT and JNK were analyzed after 30 min of HG, HG+bFGF (b) and HG+bFGF+PD173074 (PD) stimulation. All experiments were performed after 5 µg/mL mitomycin-C (cell proliferation inhibitor) application for one day. Densitometry for p-AKT (D) and p-JNK (E) was normalized to the amount of total AKT and JNK. The results are presented as fold change as compared to fibroblasts grown in the 5.5 mM glucose (LG) containing medium. Data represent mean values ±SE of three independent experiments (*P<0.05, *t* test).

Before testing HG (30 mM glucose) effects on fibroblast cell migration, effects of bFGF and FGFR1 inhibitor PD173074 were analyzed in the cell migration process. Cells were grown in an LG medium (5.5 mM glucose), and 5 µg/mL of mitomycin-C was given to cells one day prior to adding bFGF and FGFR1 inhibitor. As shown in Figure 2A and 2B, the cells treated with bFGF at 100 ng/mL had accelerated cell migration speed until 24 hours after scratching, while PD173074 supply significantly inhibited cell migration until 48 hours. This result suggests that migrating cells require activation of FGFR1 signaling, and bFGF signaling promotes fibroblast cell migration. Phosphorylation levels of AKT and JNK were reported to increase after 30 min of bFGF stimulation [5]. In human foreskin fibroblasts, p-AKT and p-JNK levels were obviously higher after 30 min in bFGF-treated cells, while inhibition of FGFR1 by PD173074 significantly repressed phosphorylation of AKT and JNK. In contrast, total AKT and JNK protein levels were not altered after bFGF and PD173074 application (Figure 2C, D, E). Further, we examined HG effects on fibroblast cell migration. To understand the specific role of HG, mannitol was used as an osmotic control. 24.5 mM mannitol was dissolved in the LG medium (5.5 mM glucose) to make a final concentration of 30 mM, identical to HG condition. Cells were grown in the LG medium for 3 days, and then transferred to HG- and mannitol-containing media and grown for another 3 days before adding bFGF or FGFR1 inhibitor. HG, not mannitol, inhibited cell migration, suggesting that HG-mediated impairment of cell migration is HG specific rather than an osmotic effect (Figure 3A and 3B). As shown in Figure 2A and 2B, bFGF promotes fibroblast cell migration; therefore, it is interesting to test whether bFGF is able to reverse HG-induced inhibition of cell migration. HG-medium grown cells treated with bFGF at 100 ng/mL showed a rescue of HG-inhibited cell migration by bFGF, but bFGF together with 50 nM PD173074 severely inhibited cell migration similar to what is shown in Figures 2A and 2B, implying that protection of bFGF on HG inhibited cell migration requires activation of FGFR1 (Figure 3A and 3B). Biochemical studies were performed to analyze cellular changes of key proteins regulated by bFGF. AKT and JNK phosphorylation was increased upon bFGF treatment as shown in Figure 2C. Subsequently, AKT and JNK phosphorylation was analyzed by Western blotting. p-AKT and p-JNK levels were decreased in HG stressed cells compared to LG feeding cells (Figure 3C, D, E). In addition, bFGF treatment partially recovered HG-suppressed p-AKT and p-JNK levels, while bFGF together with PD173074 significantly repressed p-AKT and p-JNK levels that were lower than in HG-stressed cells (Figure 3C, D, E). These results suggest that bFGF reverses HG-inhibited fibroblast cell migration as well as recovers HG-suppressed AKT and JNK phosphorylation. Furthermore, bFGF eliminates the HG effect on fibroblast cell migration in FGFR1 dependent manner.

**Figure 4:**
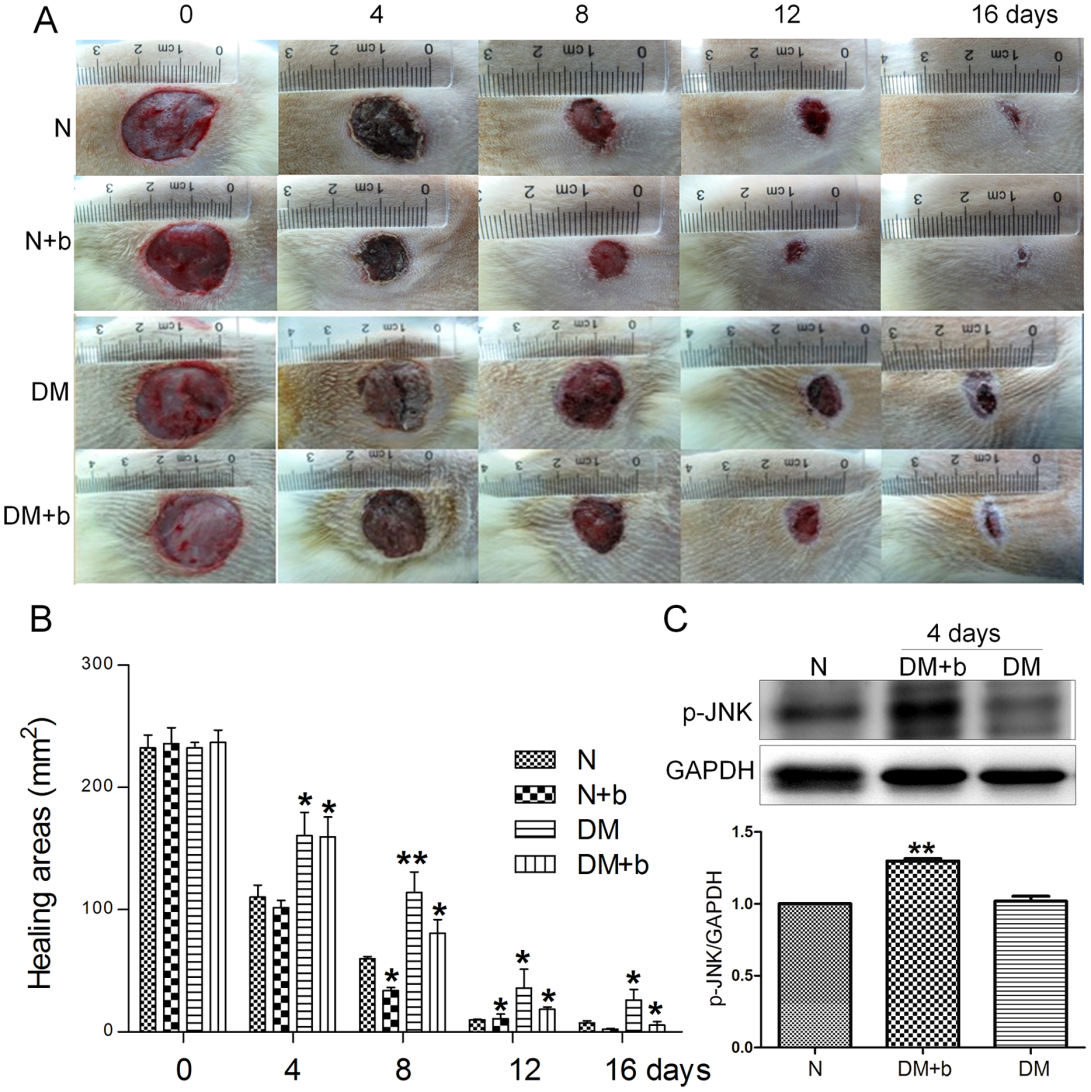
Diabetes and bFGF effects on rat skin wound repair. (A) The representative images of skin wounds from normal and diabetic rats with or without bFGF treatment. (B) Wound areas of each rat presented in (A) are counted. Wound areas are measured using TINA2.0 software (*P<0.01 t test). (C) Phosphorylation levels of JNK was analyzed after 4 days of bFGF treatment. Densitometry for p-JNK (D) was normalized to the amount of total GAPDH. The results are presented as fold change as compared to control group (N). bFGF was supplied every day. Data represent mean values ±SE of three independent experiments (**P<0.01, *t* test). N, control; N+b, control add bFGF; DM, diabetes; DM+b, diabetes add bFGF.

Diabetes is a group of metabolic diseases in which a person has high blood sugar. It is logical to ask whether bFGF medication is a treatment for diabetes-mediated skin wound repair. To understand the effects of bFGF and diabetes on skin wound repair, a type 1 diabetic rat model was established with an injection of STZ. Eight weeks after the STZ injection, two full thickness circular wounds were created on the waist of each rat and the wound repair rate was analyzed. The wound repair speed was obviously slower in diabetic rats as compared to normal rats (Figure 4A, B). bFGF was supplied every day to the wound area and its efficacy was further analyzed by monitoring the wound repair rate. The results showed that bFGF accelerates wound repair speed in both normal and diabetic rats (Figure 4A, B). Further JNK phosphorylation was analyzed from rat skin by Western blotting. The p-JNK levels were significantly higher in bFGF treated samples compared to non-diabetic or diabetic rat skin samples (Figure 4C). Taken together, bFGF efficacy on wound repair of diabetic skin is similar to bFGF protection on HG-inhibited human fibroblasts in wound healing.

### 3. HG did not alter Rac1 activity in fibroblasts

**Figure 5:**
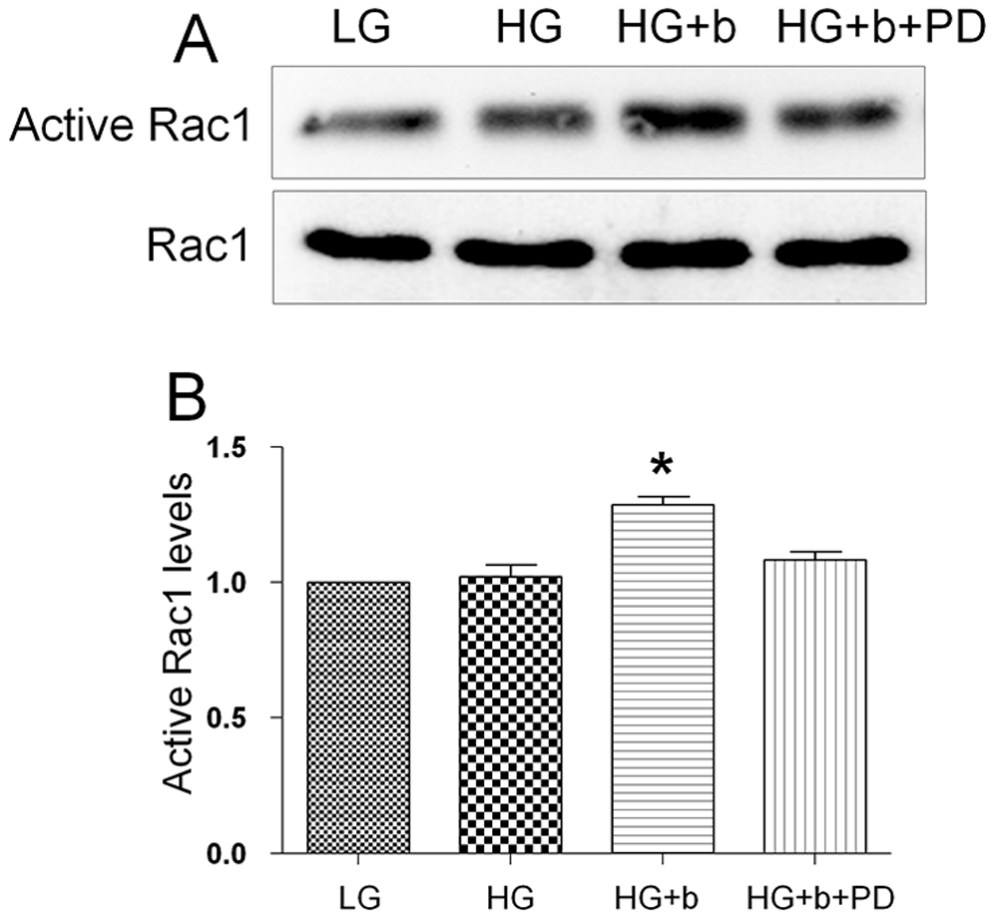
Activation of Rac1 by bFGF. (A) Activities of Rac1 were analyzed after HG, HG+bFGF (b, 100 ng/mL) and HG+b+PD173074 (PD, 50 nM) stimulation by using a pull-down system in the presence of 5 µg/mL mitomycin-C in the culture medium. (B) Densitometry for Rac1-GTP was normalized to the amount of total Rac1. The results are presented as fold change as compared to fibroblasts in the absence of bFGF. Data represent mean values ±SE of three independent experiments (*P<0.05, *t* test).

JNK is necessary for fibroblast cell migration and the PI3K-Rac1-JNK has been shown as a pathway that is downstream of bFGF to modulate cell migration in wound healing [5]. Decrease of JNK activity was observed in HG treated cells, so Rac1 was further analyzed for its activity. Rac1 is a small GTPase that plays an important role in cytoskeletal dynamics and cell migration. As shown in Figure 5, Rac1 activity was analyzed by detecting the amount of Rac1-GTP form in the cells. bFGF activates Rac1, but HG or bFGF together with PD173074 treatment to the cells did not alter Rac1 activity compared to LG medium grown cells. Together these results suggest that HG affects the downstream component of Rac1 like JNK to inhibit cell migration.

### 4. HG enhances nitrosative modification of specific proteins

**Figure 6:**
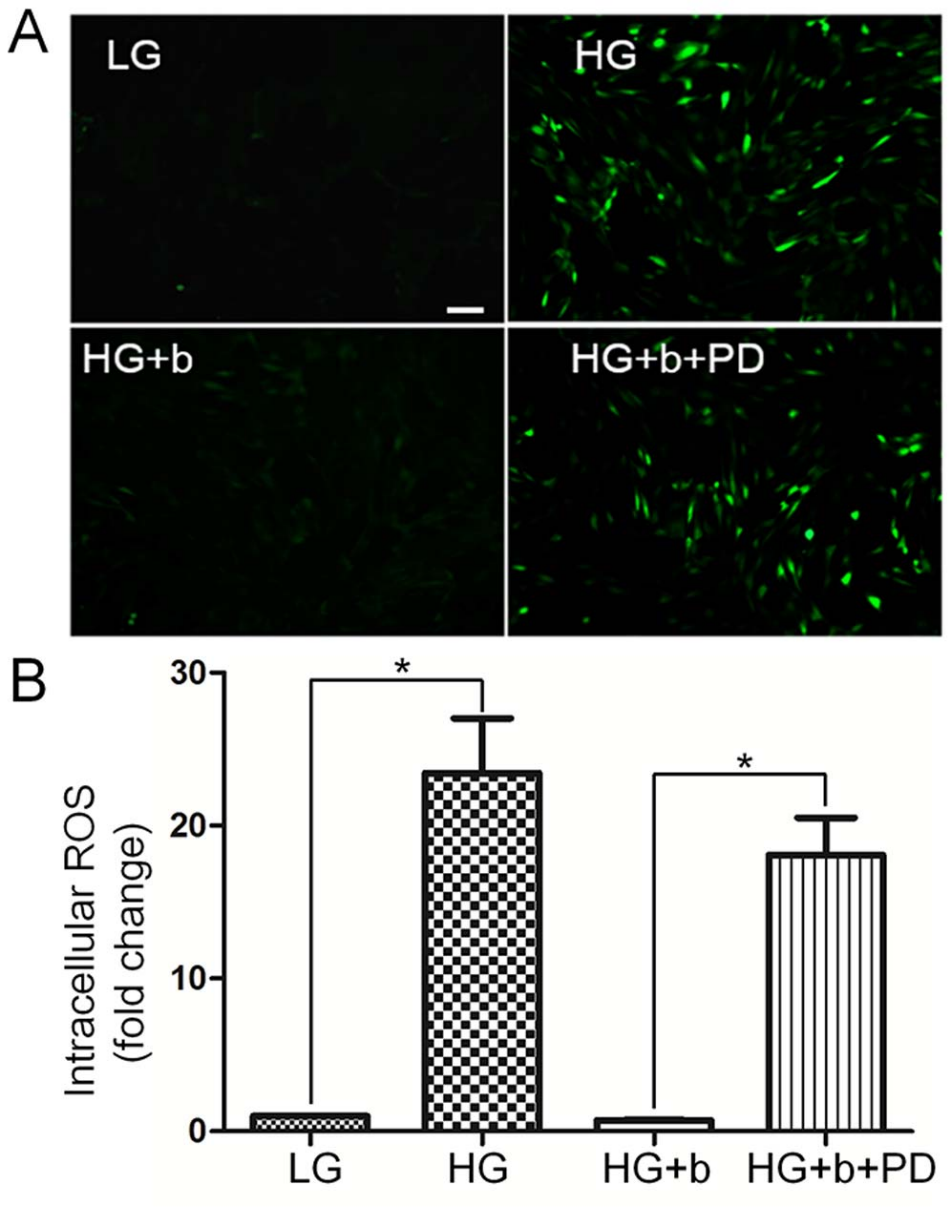
Effects of bFGF on decrease of HG-induced accumulation of intracellular ROS levels. (A) Cells were pretreated with HG (30 mM glucose) for 72 hours and then incubated with bFGF (b, 100 ng/mL) or bFGF together with FGFR1 inhibitor PD173074 (PD, 50 nM) for an additional 1 hour. LG means 5.5 mM glucose in the culture medium. Intracellular ROS levels were measured by using DCFH-DA dye. Bar = 100 µm. (B) Fluorescence levels were measured from 10 different cells in each samples using Image Pro-plus software (*n* = 10, *P<0.01 t test).

Diabetes-mediated oxidative stress is known to mainly induce different responses in tissues [20]. To test whether the HG treatment accumulates ROS, 2′, 7′-dichlorofluorescin diacetate (DCFH-DA), an indicator of ROS, was used to analyze oxidative status by monitoring fluorescence strength in the cells treated with HG, bFGF and PD173074. As shown in Figure 6, HG treatment dramatically increased ROS accumulation in the cells compared to LG medium grown cells. The bFGF supply eliminated HG-induced ROS accumulation, but bFGF with PD173074 diminished efficacy of bFGF to eliminate ROS buildup. Protein nitrations are associated with oxidative stress and lead to complications of diabetes [21], [23]. However, diabetes-induced specific protein nitrosative modifications in fibroblast cells have not been reported.

**Figure 7:**
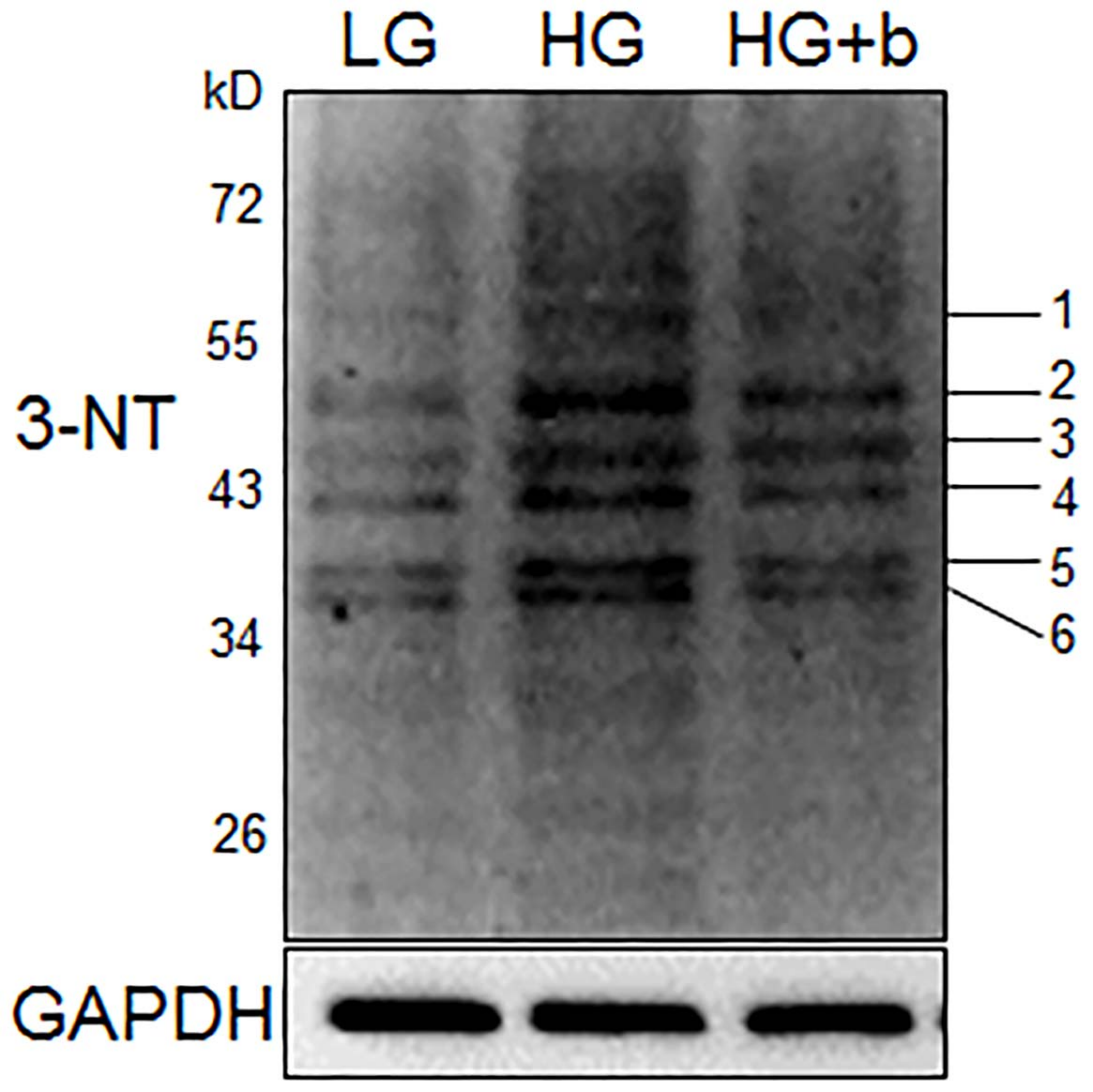
Modulation of protein nitration levels in HG and bFGF-fed fibroblast cells. Protein nitration was analyzed by immunoblotting and 3-NT antibody in HG-treated cells. bFGF (b, 100 ng/mL, 60 min) supplies repressed HG-induced increase of protein nitration levels. Numbers 1–6 on the right indicate the different nitration proteins listed in Table 1. HG and LG indicate 30 mM and 5.5 mM glucose in culture medium, respectively.

**Figure 8:**
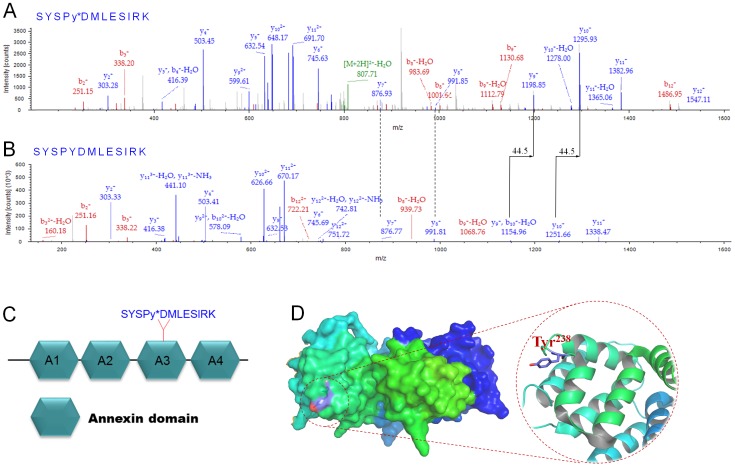
MS/MS spectra for tryptic digestion of Annexin A2 nitrated peptides. The extracted un-nitrated peptides of Annexin A2 and the corresponding nitrated peptides with +45 shift are illustrated in the top of the panel of figures for each peptide. The MS/MS spectra for the nitrated Tyr^238^ (A) and the un-nitrated Tyr^238^ (B) were shown. (C) The hexagonal indicates Annexin domain and nitration was detected at Try^238^ which is located at the third Annexin domain (A3) of Annexin A2 protein. Nitrated tyrosin residue is marked with a star. (D) Protein homology modeling of Annexin A2 using 3-D structure 1XJL as a template, Tyr^238^ located at the surface of Annexin A2.

We performed a Western blotting analysis using 3-nitrotyrosine (3-NT) antibody to determine the nitration proteins in HG-treated cells. Six proteins showed nitration levels that were significantly increased by HG, while bFGF reversed HG-induced nitration levels (Figure 7). Since protein nitration levels in the fibroblast cell are low, 3 mg of proteins extracted from HG-treated fibroblast cells were separated by electrophoresis, and each protein band was cut according to the size detected in the Western blot. Proteins further eluted from pieces of gel were used for mass spectrometry analysis. As shown in Table 1, six proteins including tubulin, actin and Annexin A2 had their nitration residues identified. Among six proteins identified, Annexin A2, a regulatory protein, was reported to promote migration and invasion of hepatocellular carcinoma cells co-cultured with fibroblasts *in vitro* [33], but its function in fibroblasts is unclear. Normally, tyrosine nitration inactivates protein due to conformational change of the protein; therefore, Annexin A2 nitration might lead to loss of its activity. We further examined the relationship between ROS and Annexin A2 nitrations to verify that nitration levels of Annexin A2 are associated with oxidative stress. The results showed that bFGF and a ROS scavenger MnTMPyP treatment decreased HG-induced Annexin A2 nitration levels, indicating that nitration levels correlate with cellular ROS levels (Figure S2). Human Annexin A2 contains 19 tyrosine residues, but interestingly only Tyr^238^ residue nitration was detected through mass spectrometry with high confidence (Figure 8A and 8B). This conclusion was based on a +44.5 mass unit shift as the typical feature of NO modification [34]. An amino acids-based domain search of Annexin A2 protein (Pfam) revealed that Annexin A2 consists of 4 Annexin domains and Tyr^238^ is located at the third Annexin domain (A3) (Figure 8C), implying that nitration at Tyr^238^ may influence the functional Annexin domain, thus inactivating Annexin A2. In addition, a 3-D view of Annexin A2 showed that Tyr^238^ is located at the surface of the protein (Figure 8D), which suggests that the Tyr^238^ residue may be easily nitrated by reactive nitrogen species. These results suggest that HG causes accumulation of ROS and protein nitrations in human fibroblasts.

**Table 1:** List of the identified nitrated peptides from different samples in SDS-PAGE with LC/MS.

Spot No.	Accession No	Description	Mr	pI	Nitrated Peptide	Ion Score	E-value	Ion Precursor	Ion Charge
1	ATPA_HUMAN	ATP synthase subunit alpha, mitochondrial	59828	9.16	K.QGQY*SPMAIEEQVAVIYAGVR.G	71	1.7e-5	2352.16	2
2	TBB4B_HUMAN	Tubulin beta-4B chain	50255	4.79	K.FWEVISDEHGIDPTGTY*HGDSDLQLER.I	43	0.02	3160.40	3
					R.SGPFGQIFRPDNFVFGQSGAGNNW*AK.G	70	4.4e-6	2843.57	3
3	ENOA_HUMAN	Alpha-enolase	47481	7.01	K.AGY*TDKVVIGMDVAASEFFR.S	42	0.0023	2286.66	3
4	ACTB_HUMAN	Actin, cytoplasmic	42052	5.29	K.DLYANTVLSGGTTMY*PGIADR.M	79	2.7e-7	2259.22	2
5	ANXA2_HUMAN	Annexin A2	38808	7.57	K.SYSPY*DMLESIRK.E	40	0.003	1632.49	2
6	G3P_HUMAN	Glyceraldehyde-3-phosphate dehydrogenase	36201	8.57	R.VIISAPSADAPMFVMGVNHEKY*DNSLK.I	37	0.0087	2977.70	3

Nitration (indicated by *) sites are shown in the identified peptide sequence. Six proteins were positively identified as nitrated proteins with the exceeded significance threshold for the required sequence database.

To test whether bFGF can inhibit diabetes-induced superoxide and 3-NT generation in the rat skin, DHE was used to stain skin tissue slices until 16 days after bFGF medication. As show in Figure S3, a DM-afflicted rat generated more superoxide than normal one, but bFGF treatment significantly reduced accumulation of superoxide in DM rat skin. Furthermore, Western blotting analysis was performed to investigate the nitrated proteins in rat skin. 3-NT levels of two proteins were increased in DM rat skin compared to normal or bFGF treated DM rat skin (Figure S4). The proteins were further identified by a mass spectrometry analysis, and the data indicate that the nitrated proteins are succinyl-CoA:3-ketoacid CoA transferase-1 (SCOT) and ATP synthase α subunit. These results suggest that DM cause accumulation of superoxide and nitration proteins while bFGF has protective effects on DM rat skin.

### 5. JNK inhibition leads to the increase of Annexin A2 nitration and impairs cell migration

**Figure 9:**
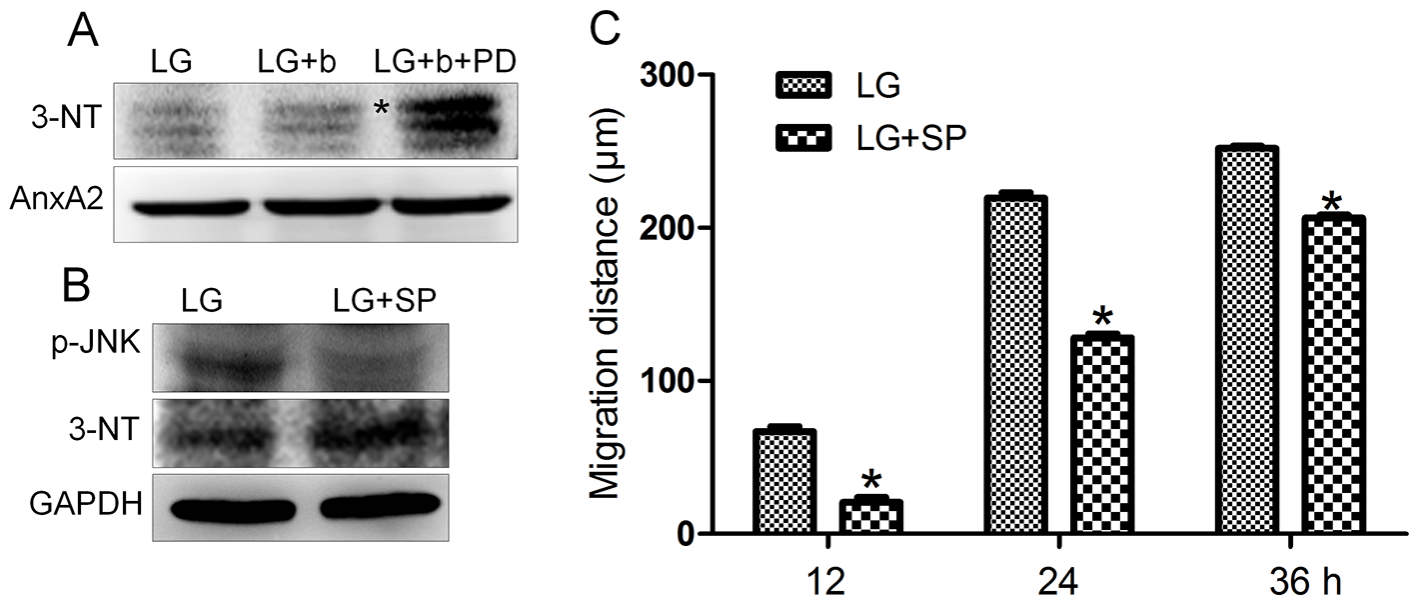
Effects of inhibition of FGFR1 and JNK on Annexin A2 nitration. (A) The Annexin A2 nitration was analyzed by immunoblotting with treatment of bFGF (b, 100 ng/mL, 30 min) or FGFR1 inhibitor PD173074 (PD, 50 nM, 30 min) under LG growth conditions (5.5 mM glucose). 3-NT and Annexin A2 antibodies were used to detect the nitrated and total Annexin A2 protein, respectively. The asterisk indicates nitrated Annexin A2. (B) The p-JNK and Annexin A2 nitration levels with or without SP600125 (SP, 25 µM, 60 min) were analyzed by immunoblotting. GAPDH protein was used as a control for normalizing of loading. (C) Analysis of the migration rate of JNK-inhibited cells was performed. Data represent mean values ±SE of 10 replications (*P<0.001, *t* test).

**Figure 10:**
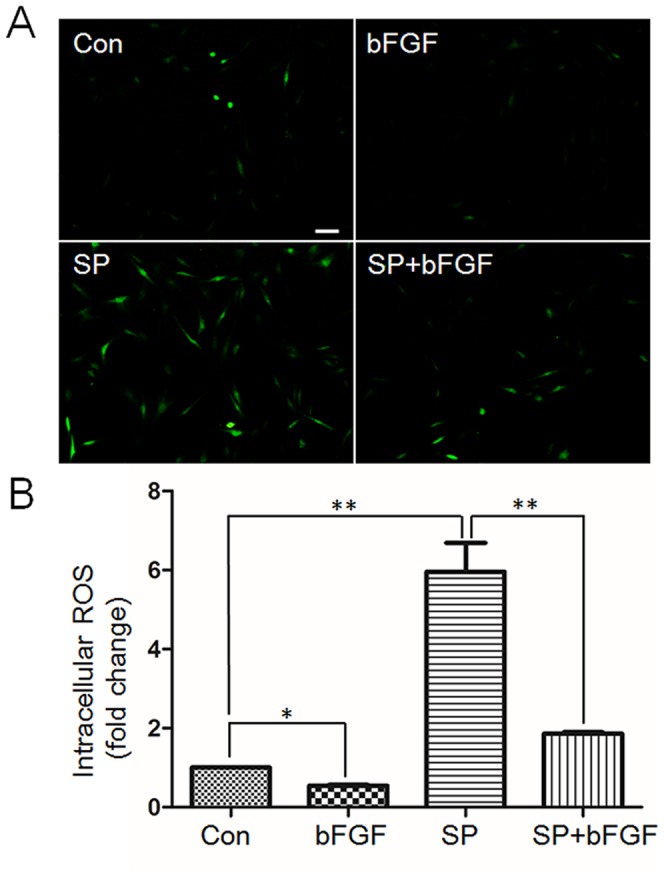
Effects of SP600125 and bFGF on cellular ROS accumulation. (A) Cells were incubated with bFGF (b, 100 ng/mL), JNK inhibitor SP600125 (SP, 25 µM) or bFGF together with SP600125 for 1 hour. All experiments were performed in low glucose (5.5 mM) containing medium. Intracellular ROS levels were measured using DCFH-DA dye. Bar = 100 µm. (B) Fluorescence levels in 10 different cells from each samples shown in (A) were measured using Image Pro-plus software (*n* = 10, *P<0.01 t test).

**Figure 11:**
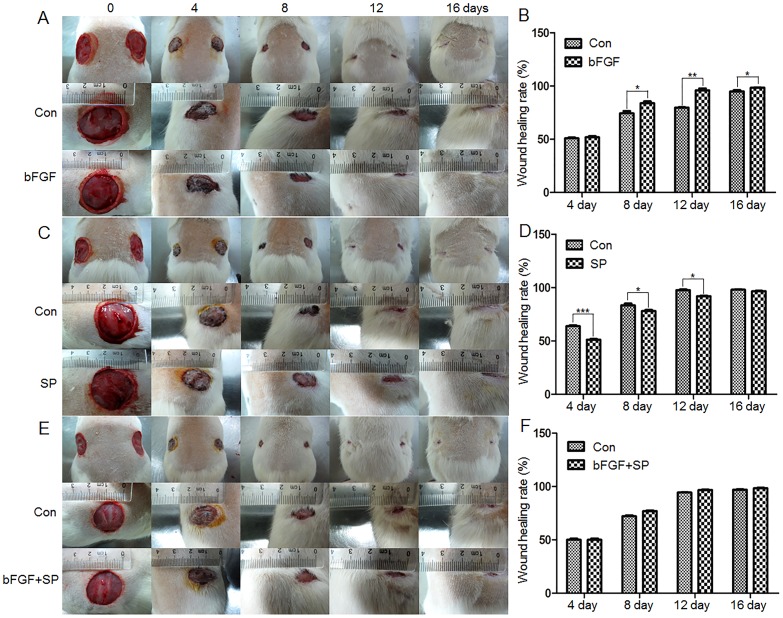
bFGF and SP600125 effects on rat skin wound repair. (A, B) Skin wounds from normal rats with or without bFGF treatment. (C, D) Skin wounds from normal rats with or without JNK inhibitor SP600125 treatment. (E, F) Skin wounds from normal rats treated with or without JNK inhibitor SP600125 together with bFGF. bFGF was supplied every day. Data represent mean values ±SE of three independent experiments (**P<0.01, *t* test). Wound healing was monitored until 16 days after the treatment. Wound areas are measured using TINA2.0 software. N, control; N+b, control add bFGF; DM, diabetes; DM+b, diabetes add bFGF.

bFGF reversed HG inhibited JNK phosphorylation and increased nitration levels of specific proteins, such as Annexin A2 (Figure 3C and 7). Experiments were performed to understand the relationship between JNK activity and Annexin A2 nitration. PD173074 and SP600125 are known to inhibit FGFR1 and JNK, respectively, and were supplied to fibroblasts. PD173074 treatment increased nitration levels of Annexin A2, while total Annexin A2 levels were not changed (Figure 9A). SP600125 application suppressed p-JNK, but increased Annexin A2 nitration levels (Figure 9B). Further, application of PI3K inhibitor LY294002 drastically suppressed AKT and JNK phosphorylation levels in fibroblasts (Figure S5). JNK inhibitor SP600125 partially inhibited bFGF protective effects on HG-regulating protein nitrations, suggesting that bFGF action on protein nitration is partially via JNK (Figure S6 and S7). However, 5 proteins out of 6 proteins were detected whose nitrations were regulated by JNK (Figure S6 and S7), indicating that one protein's nitration is independent of JNK pathway and regulated by bFGF signaling. In addition, SP600125 treatment significantly delayed fibroblast cell migration speed (Figure 9C). Because application of JNK inhibitor SP600125 increased protein nitration levels; therefore, intracellular ROS levels were monitored after treatment of SP600125 or SP600125 together with bFGF. The results showed that SP600125 supply significantly increased cellular ROS levels while bFGF treatment blocked SP600125 effects on ROS accumulation in the fibroblast (Figure 10). Effects of SP600125 were also analyzed in rat model. SP600125 application obviously delayed skin wound healing speed in both normal and bFGF medicated rats (Figure 11). Taken together, these results suggest that JNK acts at downstream of bFGF signaling, is a key regulator in fibroblast cell migration, and bFGF may repress Annexin A2 nitration levels via activation of JNK in human fibroblasts cells.

## Discussion

Cell migration and proliferation are important processes that trigger new extracellular matrix synthesis and contribute to wound healing [35]. HG effects on cell proliferation were analyzed with 10% or 0.35% FBS in the culture media. HG did not visibly affect cell proliferation with 10% FBS, but HG significantly inhibited cell proliferation even under the concentration of 15 mM with 0.35% FBS in the culture medium. In 10% FBS, the fibroblast cells were more resistant to environmental stress than in 0.35% FBS. HG was reported to increase rat fibroblast cell size [18], but we did not observe obvious morphological differences in HG treated human fibroblast cells (data not shown). However, HG inhibited cell migration with a delay of migrating speed that is similar with the delay in rat fibroblasts (Figure 3A), [18].

bFGF promotes cell migration and proliferation in the wound healing process. A recent study reported that bFGF regulates the PI3K-Rac1-JNK pathway to promote cell migration in fibroblasts [5]. Biochemical studies demonstrated that HG suppresses JNK phosphorylation, and adding bFGF reversed the HG effects on JNK activation (Figure 3C). Further experiments showed that bFGF, not HG, activated Rac1 in human fibroblasts (Figure 5). In CHO.K1 cells, HG activated Rac1 and inhibited cell migration, implying that there are different regulation processes of Rac1 in human and mouse for HG-stress and activity in cell migration. PI3K, Rac1 and JNK inhibition impaired cell migration, and our data indicate that HG inhibits cell migration via suppression of p-JNK level rather than Rac1 activity in human foreskin fibroblasts. PI3K activity has not been analyzed in our study due to normal activity of Rac1 that was revealed in human fibroblasts upon HG stress. However, it may be interesting to analyze PI3K activity after HG stress to understand whether another pathway is able to regulate JNK from PI3K that is independent of Rac1 in human fibroblasts. JNK (c-Jun N-terminal kinase) was originally identified as a kinase that binds and phosphorylates c-Jun protein at its transcriptional activation domain. JNK belongs to the mitogen-activated protein kinase (MAPK) family, and are responsive to cytokines, ultraviolet irradiation, heat, and osmotic stresses [36]. Numerous stresses, inflammatory signals, ultraviolet radiation, and protein synthesis inhibitors change ROS levels and activate JNK [37], indicating that JNK is activated by environmental damage to the cells. HG caused an increase in cellular ROS levels, and also inhibited JNK phosphorylation (Figure 3C and 6). Furthermore, bFGF activates JNK to promote cell migration in fibroblasts. Application of JNK inhibitor into fibroblasts significantly inhibited cell migration (Figure 9C), suggesting that the roles of JNK activation varies in response to different stimuli.

Usually, many symptoms are associated with ROS and protein nitration levels in diabetic tissues [20]. The formation of 3-nitrotyrosine (3-NT) is resulted from the overproduction of superoxide that in turn reacts with nitric oxide to produce peroxynitrite (ONOO^−^). ONOO^−^ is a reactive oxidant with a short half-life time and able to modify several amino acid residues, such as tyrosine, cysteine, tryptophan and methionine. Tyrosine nitration changes the structure and function of the proteins, and its correlations with diseases were reported in several studies [34], [38]. Our results revealed that cellular ROS and protein nitration were greatly enhanced either in HG treated fibroblasts (Figure 6 and 7) or in diabetic rat skin (Figure S3 and S4). Six proteins with increases of nitration levels were further discovered, and nitration sites in each protein were identified using mass spectrometry analysis (Table 1). Interestingly, cytoskeleton proteins like tubulin and actin were seen, and we also discovered the location of nitration sites within regulatory domains that greatly increased the possibility of inactivation of tubulin and actin proteins by nitrosative modifications. A previous study reported that actin proteins are key regulators in cell migration steps. Rac1, a GTPase, activates the actin-mediated Wave complex to induce the formation of lamellipodial protrusions at the leading edge of migrating cells [9]. HG did not affect Rac1 activity in human fibroblasts, and one actin cytoplasmic 1 protein nitration was identified. Further experiments are required to test the role of nitration at actin cytoplasmic 1, and find a novel regulatory mechanism of actin proteins under diabetes. Among them, Annexin A2 was identified by a Tyr^238^ nitration that was located at the third Annexin domain (Figure 8). Annexin A2 was reported to regulate glioma cell invasion, tumor progression, migration and invasion of human hepatocellular carcinoma cells as well as intestinal epithelial cell migration [33], [39], [40]. In addition, Tyr^23^ phosphorylation is important to Annexin A2, which binds the endosome [41]. With these findings, we postulate that Annexin A2 nitration at Tyr^238^ residue might change Annexin A2 activity and cell migration in human fibroblasts. It is important to note that DM causes SCOT and ATP synthase α protein nitration, indicating that the targets of nitrated protein by HG or DM are different in humans and rats.

JNK is a key regulator in cell migration and its inactivation inhibited cell migration. Interestingly, FGFR1 and JNK inhibitor treatment increased Annexin A2 nitration and cellular ROS levels (Figure 9B and 10) and bFGF reversed an HG mediated increase of Annexin A2 nitration (Figure 7, S6 and S7). Furthermore, JNK activation by bFGF was suppressed by inhibition of FGFR1 (Figure 2). Our data implies that bFGF inhibits Annexin A2 nitration may be via activation of JNK in human fibroblast cells. This is the first study for understanding the molecular mechanism of HG-inhibited human fibroblast cell migration. We showed here that HG repressed bFGF regulated JNK phosphorylation and increased specific protein nitration. Further, JNK inactivation enhanced protein nitration that might result in a delay of cell migration. We propose that JNK inactivation may cause impairment of wound healing in diabetic skin. Importantly, our results have significance beyond the laboratory. In humans as in mice, bFGF medication could be a possible treatment for diabetes mellitus (DM), pending further studies.

## Supporting Information

Figure S1 **Effects of HG and bFGF on the cell proliferative rates.** HSFs treated with indicated concentrations of bFGF for up to 48, 72 and 96 hours after 30 mM glucose stimulation and then cell proliferation was measured by CCK-8 assay. Data indicate mean values ±SE of five independent experiments, as compared to the control group (*P<0.05, *t* test).
(TIF)

Figure S2 **Effects of bFGF and MnTMPyp on HG-induced nitration of Annexin A2.** Annexin A2 levels were analyzed by immunoblotting (A) with supplementation of bFGF (100 ng/ml, 60 min) or (B) with the application of 50 µM metalloporphyrin- based superoxide dismutase (MnTMPyP, 60 min).
(TIF)

Figure S3 **bFGF inhibits superoxide accumulation in diabetic rat skin.** Skin tissues from Normal (N), DM and DM+bFGF (b, 100 ng/mL) were examined under the light microscope after DHE staining for superoxide followed by semi-quantitative analysis. bFGF was applied every day. Bar = 100 µm.
(TIF)

Figure S4 **Modulation of protein nitration levels in diabetic and bFGF-medicated rat skin.** Protein nitration was analyzed by immunoblotting with 3-NT antibody. bFGF (b, 90 U/cm^2^) supplies repressed DM-induced increase of protein nitration levels. Numbers 1 (succinyl-CoA:3-ketoacid CoA transferase-1) and 2 (ATP synthase α subunit) on the right indicate the different nitration proteins. bFGF was applied every day.
(TIF)

Figure S5 **Effects of PI3K inhibitor on AKT and JNK phosphorylation in fibroblasts.** Phosphorylation levels of AKT and JNK proteins were analyzed 60 min after LY294002 (LY, PI3K inhibitor, 10 µM) stimulation. All experiments were performed after 5 µg/mL mitomycin-C (cell proliferation inhibitor) application for one day. LG means 5.5 mM glucose.
(TIF)

Figure S6 **Modulation of protein nitration levels in HG, bFGF and JNK inhibitor treated fibroblast cells.** Protein nitration was analyzed by immunoblotting and 3-NT antibody in HG-treated cells. bFGF (b, 100 ng/mL, 60 min) supplies repressed HG-induced increase of nitration levels and JNK inhibitor SP600125 (SP, 25 µM, 60 min) reverses it partly. Numbers 1–6 on the right indicate the different nitrated proteins listed in Table 1. HG and LG indicate 30 mM and 5.5 mM glucose in culture medium.
(TIF)

Figure S7 **Densitometry for modificatory of protein nitration levels shown in Figure S6.** Protein nitration was analyzed by immunoblotting and 3-NT antibody in HG treated cells. bFGF (b, 100 ng/mL, 60 min) supplies repressed HG-induced increase of protein nitration levels and JNK inhibitor SP600125 (SP,25 µM,60 min) reverses it partly. HG and LG indicate 30 mM and 5.5 mM glucose in culture medium. Densitometry for protein ATPA (A) or TBB4B (B) or ENOA (C) or ACTB (D) or ANXA2 (E) or G3P (F) was nearly normalized to the amount of total GAPDH. The results are presented as fold change as compared with control group (N). Data represent mean values ±SE of three independent experiments (*P<0.05,**P<0.01, *t* test).
(TIF)
