# Supplementary figures and images for: High-Glucose Inhibits Human Fibroblast Cell Migration in Wound Healing via Repression of bFGF-Regulating JNK Phosphorylation
Source: PLoS One. 2014 Sep 22;9(9):e108182. doi: 10.1371/journal.pone.0108182 (PMC4171528; doi:10.1371/journal.pone.0108182)

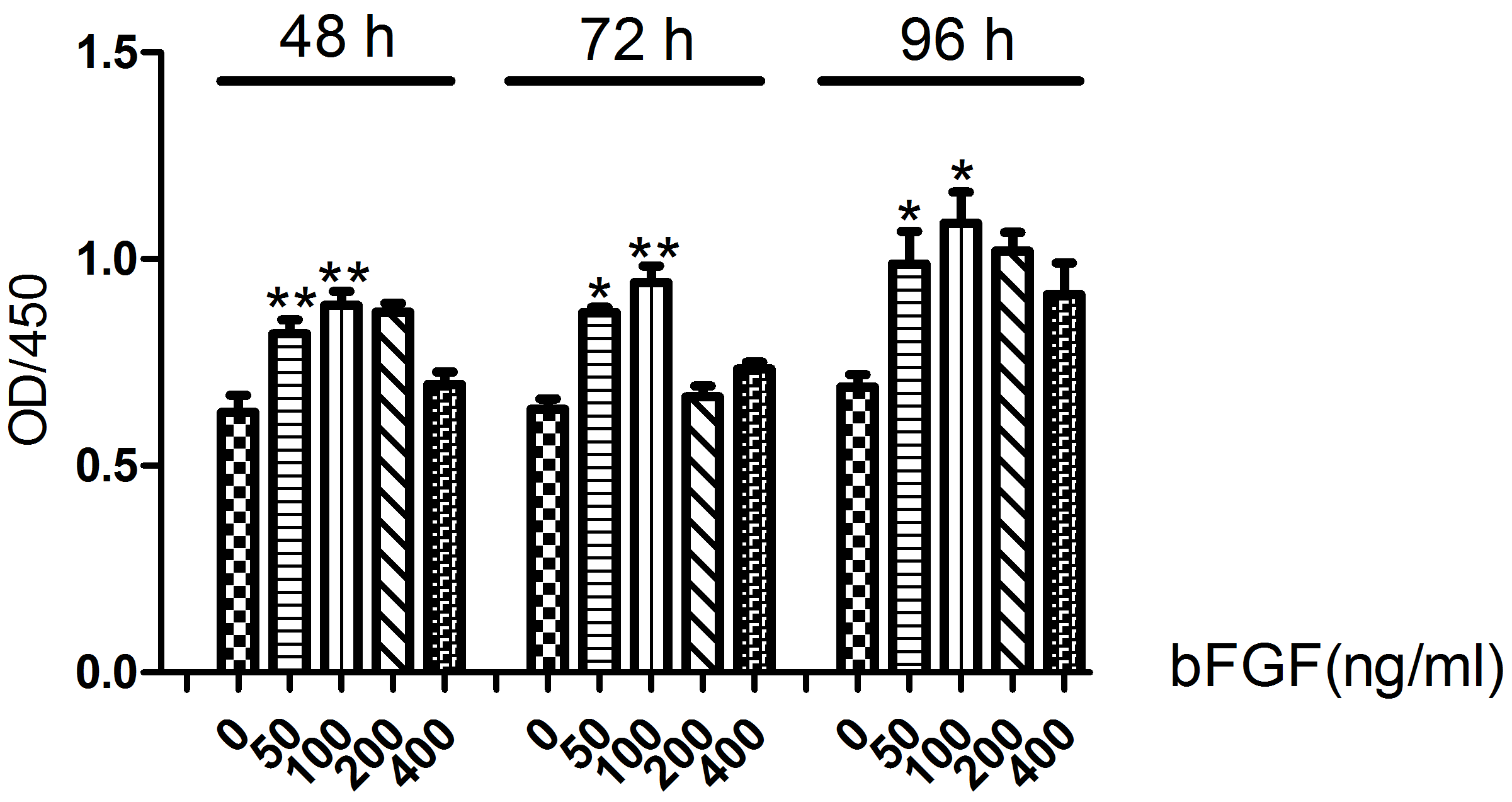

Supplement: Figure S1 — Effects of HG and bFGF on the cell proliferative rates. HSFs treated with indicated concentrations of bFGF for up to 48, 72 and 96 hours after 30 mM glucose stimulation and then cell proliferation was measured by CCK-8 assay. Data indicate mean values ±SE of five independent experiments, as compared to the control group (*P<0.05, t test). (TIF) [file pone.0108182.s001.tif]

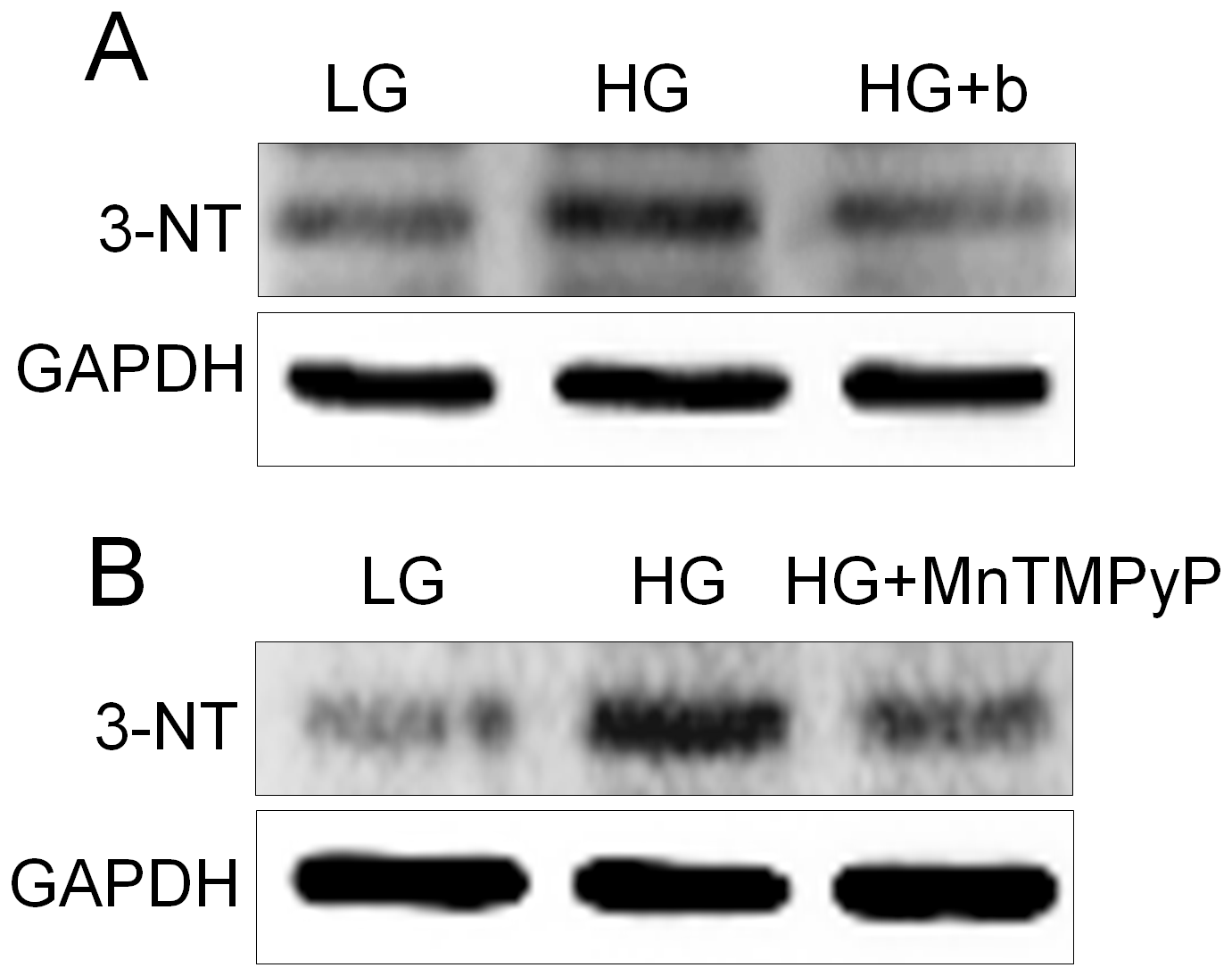

Supplement: Figure S2 — Effects of bFGF and MnTMPyp on HG-induced nitration of Annexin A2. Annexin A2 levels were analyzed by immunoblotting (A) with supplementation of bFGF (100 ng/ml, 60 min) or (B) with the application of 50 µM metalloporphyrin- based superoxide dismutase (MnTMPyP, 60 min). (TIF) [file pone.0108182.s002.tif]

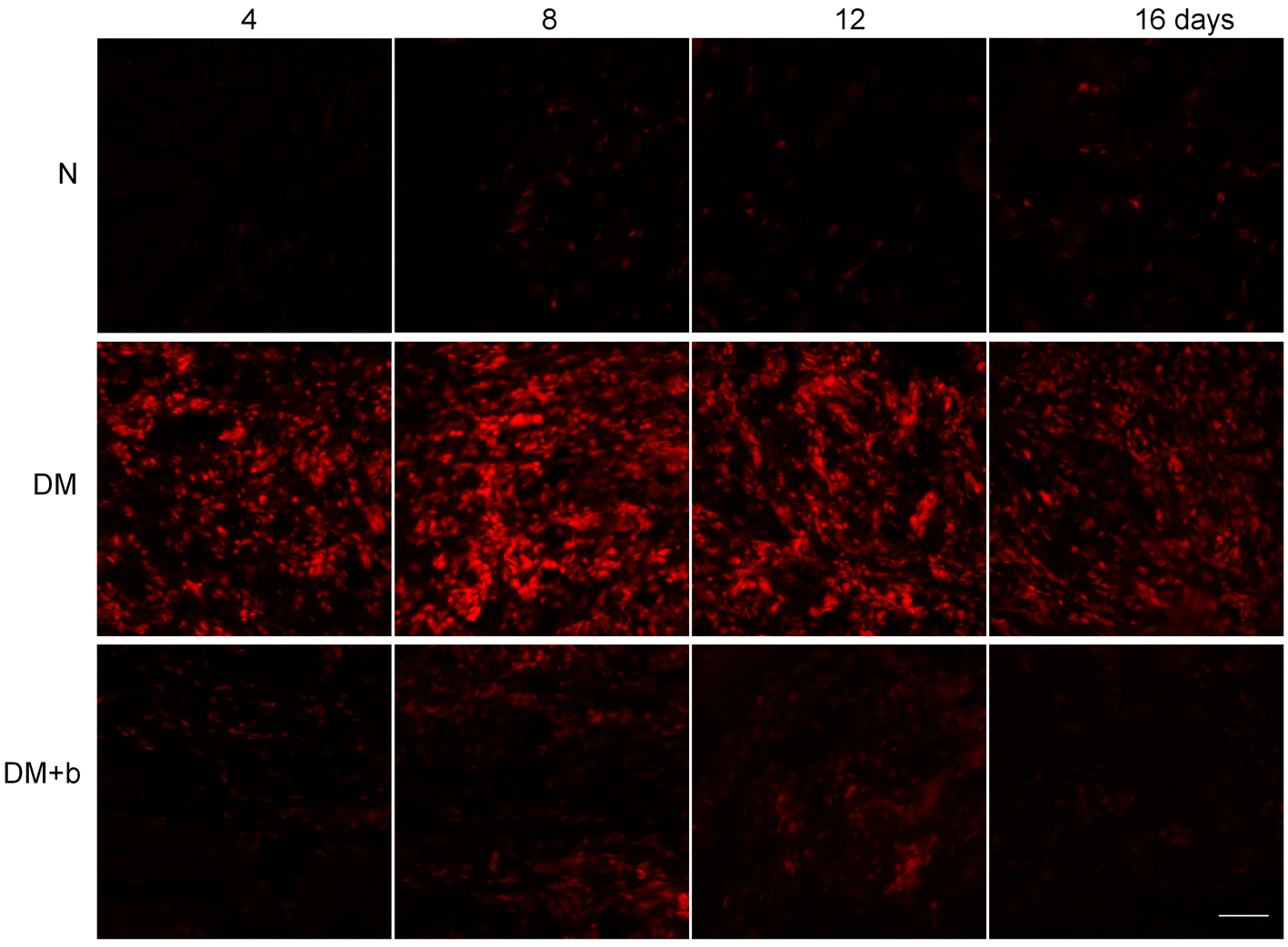

Supplement: Figure S3 — bFGF inhibits superoxide accumulation in diabetic rat skin. Skin tissues from Normal (N), DM and DM+bFGF (b, 100 ng/mL) were examined under the light microscope after DHE staining for superoxide followed by semi-quantitative analysis. bFGF was applied every day. Bar = 100 µm. (TIF) [file pone.0108182.s003.tif]

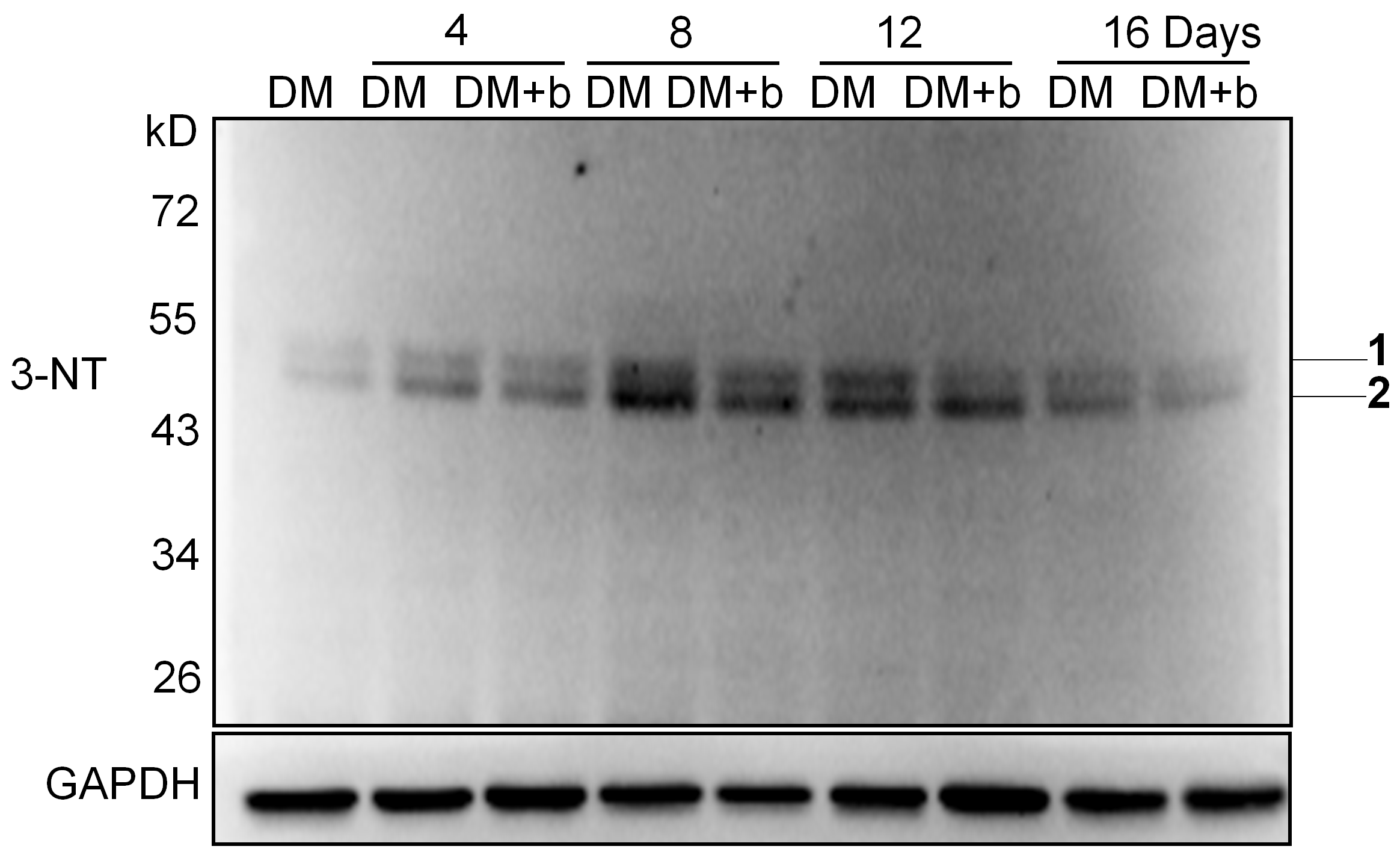

Supplement: Figure S4 — Modulation of protein nitration levels in diabetic and bFGF-medicated rat skin. Protein nitration was analyzed by immunoblotting with 3-NT antibody. bFGF (b, 90 U/cm2) supplies repressed DM-induced increase of protein nitration levels. Numbers 1 (succinyl-CoA:3-ketoacid CoA transferase-1) and 2 (ATP synthase α subunit) on the right indicate the different nitration proteins. bFGF was applied every day. (TIF) [file pone.0108182.s004.tif]

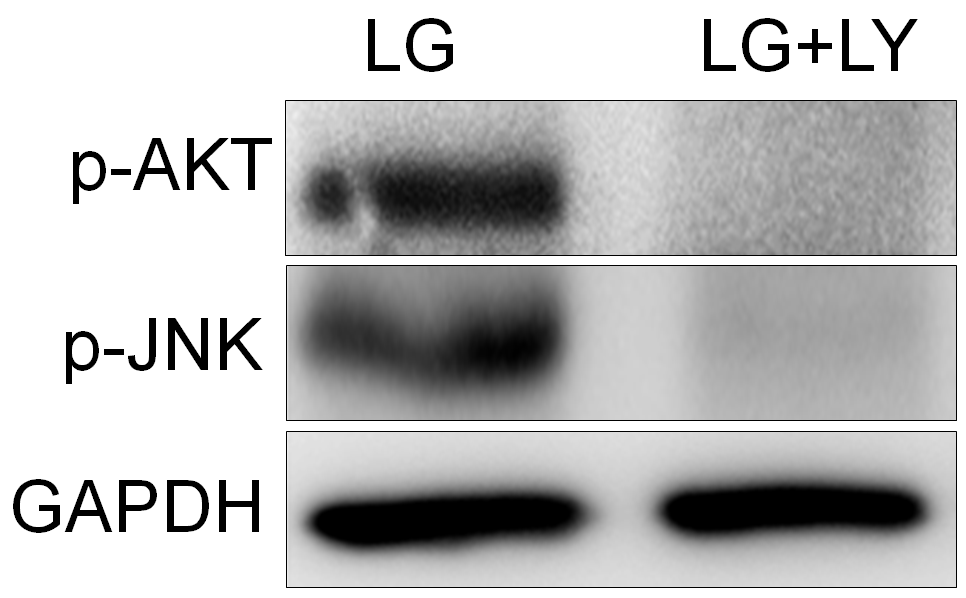

Supplement: Figure S5 — Effects of PI3K inhibitor on AKT and JNK phosphorylation in fibroblasts. Phosphorylation levels of AKT and JNK proteins were analyzed 60 min after LY294002 (LY, PI3K inhibitor, 10 µM) stimulation. All experiments were performed after 5 µg/mL mitomycin-C (cell proliferation inhibitor) application for one day. LG means 5.5 mM glucose. (TIF) [file pone.0108182.s005.tif]

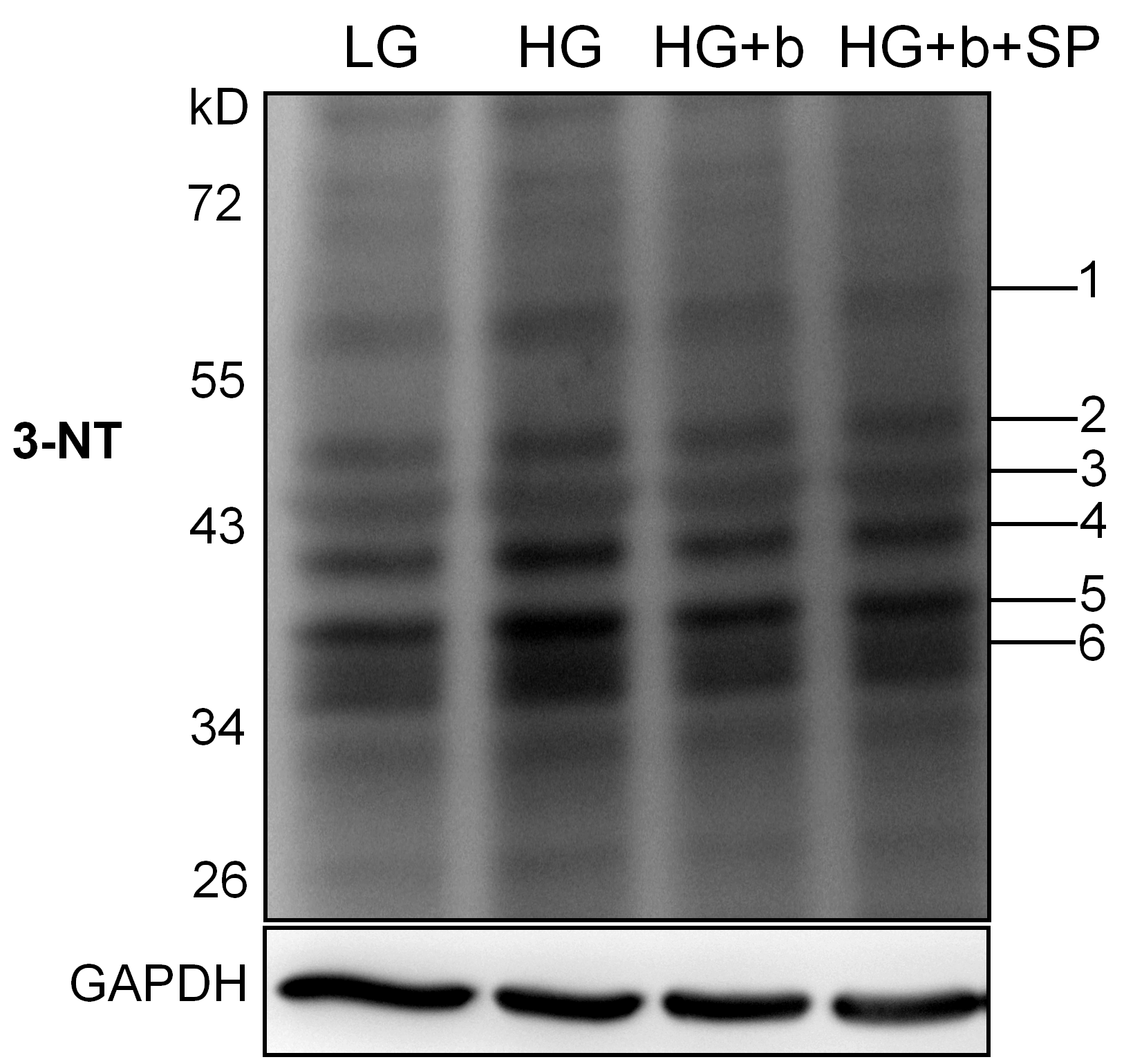

Supplement: Figure S6 — Modulation of protein nitration levels in HG, bFGF and JNK inhibitor treated fibroblast cells. Protein nitration was analyzed by immunoblotting and 3-NT antibody in HG-treated cells. bFGF (b, 100 ng/mL, 60 min) supplies repressed HG-induced increase of nitration levels and JNK inhibitor SP600125 (SP, 25 µM, 60 min) reverses it partly. Numbers 1–6 on the right indicate the different nitrated proteins listed in Table 1. HG and LG indicate 30 mM and 5.5 mM glucose in culture medium. (TIF) [file pone.0108182.s006.tif]

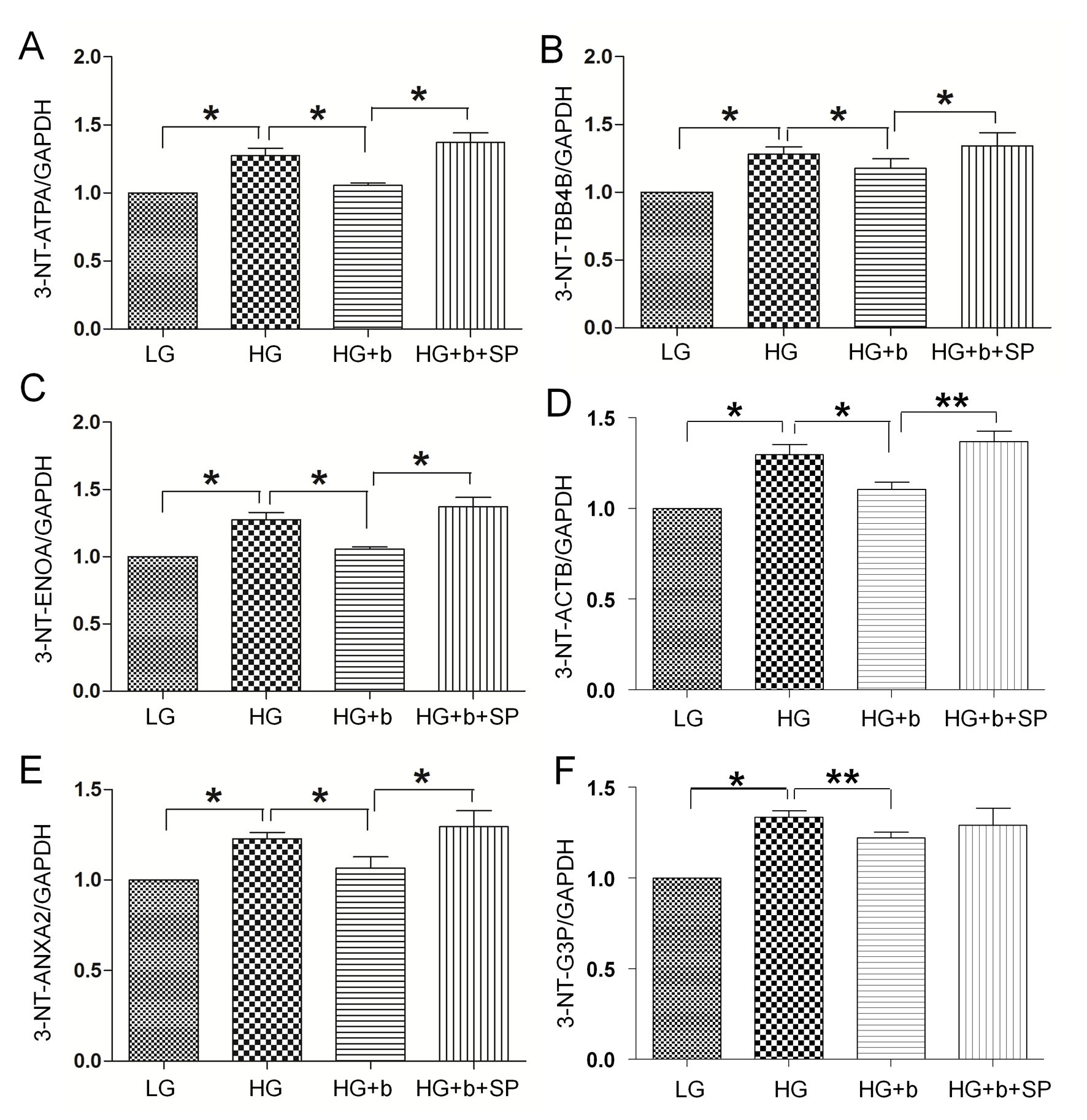

Supplement: Figure S7 — Densitometry for modificatory of protein nitration levels shown in Figure S6. Protein nitration was analyzed by immunoblotting and 3-NT antibody in HG treated cells. bFGF (b, 100 ng/mL, 60 min) supplies repressed HG-induced increase of protein nitration levels and JNK inhibitor SP600125 (SP,25 µM,60 min) reverses it partly. HG and LG indicate 30 mM and 5.5 mM glucose in culture medium. Densitometry for protein ATPA (A) or TBB4B (B) or ENOA (C) or ACTB (D) or ANXA2 (E) or G3P (F) was nearly normalized to the amount of total GAPDH. The results are presented as fold change as compared with control group (N). Data represent mean values ±SE of three independent experiments (*P<0.05,**P<0.01, t test). (TIF) [file pone.0108182.s007.tif]
